# Effect of Four Novel Bio-Based DES (Deep Eutectic Solvents) on Hardwood Fractionation

**DOI:** 10.3390/molecules25092157

**Published:** 2020-05-05

**Authors:** Paulo Torres, Mercè Balcells, Enrique Cequier, Ramon Canela-Garayoa

**Affiliations:** Department of Chemistry-DBA center, University of Lleida, ETSEA, Av. Alcalde Rovira Roure 191, 25198 Lleida, Spain; pctorresp@quimica.udl.cat (P.T.); balcells@quimica.udl.cat (M.B.); ecequier@quimica.udl.cat (E.C.)

**Keywords:** choline chloride (ChCl), deep eutectic solvent (DES), extraction processes, holocellulose, hydrogen-bond acceptor (HBA), hydrogen-bond donor (HBD), ionic liquid (IL), lignin, lignocellulosic materials

## Abstract

Using the basic principle of construction between a hydrogen bond acceptor (HBA) and a hydrogen bond donor (HBD), four bio-based deep eutectic solvents (DESs) were prepared in a 1:2 molar ratio of HBA:HBD. 2,3-Dihydroxypropyl-1-triethylammonium chloride ([C_9_H_22_N^+^O_2_]Cl^−^) was synthesized from raw glycerol and used as an HBA. Lactic acid, urea, pure glycerol, and ethylene glycol were selected as HBD. Attempts to prepare DESs, using citric acid and benzoic acid as HBDs, were unsuccessful. All these DESs were characterized using FTIR and NMR techniques. Besides, physicochemical parameters such as pH, viscosity, density, and melting point were determined. The behavior of these DES to fractionate olive pomace was studied. Lignin recovery yields spanned between 27% and 39% (*w*/*w*) of the available lignin in olive pomace. The best DES, in terms of lignin yield ([C_9_H_22_N^+^O_2_]Cl^−^ -lactic acid), was selected to perform a scale-up lignin extraction using 40 g of olive pomace. Lignin recovery on the multigram scale was similar to the mg scale (38% *w*/*w*). Similarly, for the holocellulose-rich fractions, recovery yields were 34% and 45% for mg and multi-gram scale, respectively. Finally, this DES was used to fractionate four fruit pruning samples. These results show that our novel DESs are alternative approaches to the ionic liquid:triethylammonium hydrogen sulfate and the widely used DES: choline chloride:lactic acid (1:10 molar ratio) for biomass processing.

## 1. Introduction

The biomass of a plant origin is mostly composed of lignocellulosic material, which represents an abundant and cost-effective source in the world of renewable organic compounds [1]. Lignocellulosic material consists mainly of three different types of polymers: cellulose, hemicellulose, and lignin. Lignin is the only aromatic biopolymer in nature [2], whereas the other two are polysaccharides [3].

Deep eutectic solvents (DES) are eutectic liquids with similar characteristics to ionic liquids (IL), but with marked environmental, economic, and synthetic advantages [4]. DESs are defined as a mixture between a hydrogen bond acceptor (HBA), often a quaternary ammonium salt, and a hydrogen bond donor (HBD), which can be alcohols, acids, amines, or carbohydrates among others [5,6]. DESs share several physical properties with the widely studied ILs such as low vapor pressure, low melting point, relatively wide liquid range, non-flammability, and tunability (depending on the peculiarities of the reactions) [7]. In contrast, DESs present some chemical differences, e.g., IL are molten salts formed by a discrete anion and cation type, whereas DESs are systems formed from eutectic mixtures containing a variety of cationic and anionic species [8], creating a charge delocalization between anion and the HBD compounds [9]. Although hydrogen bonds have different contact distances and binding energies, the properties of DESs do not depend only on the nature of the donor and the acceptor [6]. A wide range of DESs can be built by an appropriate selection of their components, as well as their stoichiometric ratios. There is an extensive molecular library of HBDs and HBAs used in the preparation of DESs [6].

DESs are widely used in the fractionation of biomass. Recent documents describe a lignin extraction of 14.90 wt% using malic acid:proline 3:1, while 6.48 wt% of cellulose dissolution was achieved using oxalic acid:allyltriethylammonium chloride (1:1) [10,11,12]. Moreover, DESs have been used in processes such as chitin dissolution and chitin nanofiber preparation, starch processing, rice straw pretreatment [9], and soybean oil extraction [13]. Furthermore, DESs also have wide applications in different fields, such as CO_2_ entrapment, catalysis, electrochemistry, and nanomaterial preparation [7].

**Figure 1:**
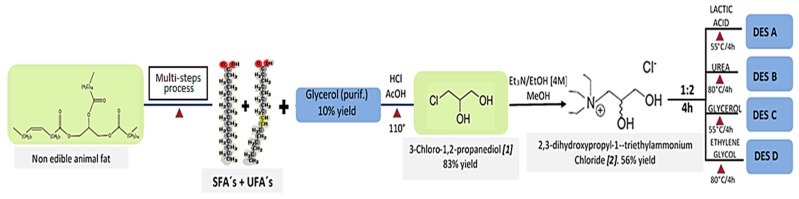
The process to prepare four novel bio-based DESs.

The present document aims to describe the synthesis of [C_9_H_22_N^+^O_2_]Cl^−^ (DPTAC) (**2**) and the preparation of new DESs using compound **2** as the HBA and four HBD compounds (Figure 1). These DESs have not been described until now, to our knowledge. Moreover, these DES were tested in the fractionation of hardwood material to deepen the understanding of their behavior as green solvents in the fractionation of biomass. Finally, the behavior of these DES was compared with the results obtained using the widely used choline chloride-lactic acid (ChCl:LA 1:10 molar ratio) and triethylammonium hydrogen sulfate([Et_3_NH] [HSO_4_] (1:1)) solvents.

## 2. Results and Discussion

A 1:2 mixture of DPTAC with glycerol, lactic acid, urea or ethylene glycol led to [DPTAC][Gly], [DPTAC][LA], [DPTAC][Urea], and [DPTAC][Eg] deep eutectic solvents, respectively. After confirming that the homogenous mixtures were stable after 24 h of agitation, the formation of DESs was confirmed by ^1^H NMR and FT-IR spectroscopies. ^1^H NMR chemical shifts of the eutectic mixture were compared with the chemical shifts of each of the precursors. Table S1 and Table S2 show the peak assignments from NMR and IR bands before and after the formation of the eutectic mixture (see Supplementary Material). Due to chemical interactions and the effect of the hydrogen bonds, the signals of compound **2** in the DESs showed different chemical shifts concerning the NMR of the compound.

Similarly, FT-IR spectroscopy was used to monitor the formation of the eutectic mixtures between 4000–3200 cm^−1^. The OH band of **2** overlapped with the OH band of glycerol, lactic acid, and ethylene glycol present in [DPTAC][Gly], [DPTAC][LA], and [DPTAC][Eg] respectively. However, the intensity of these OH bands in DESs was lower. The characteristic C=O band of lactic acid and urea (1730 cm^−1^) was shifted to a lower wavelength number in eutectic mixtures [DPTAC][LA] and [DPTAC][Urea] (see Supplementary data).

### 2.1. Physicochemical Properties

Table 1 shows the density (δ) at 25 °C, pH, and viscosity (η) of the DESs and IL considered in this study. These DESs described are viscous liquids at 23 °C (except [DPTAC][Urea], which is a solid at room temperature due to an extensive hydrogen-bonding network resulting in high viscosity. Furthermore, bio-based DES described are mainly soluble/miscible in polar solvents (e.g., methanol, dimethyl sulfoxide (DMSO), and water).

Table 1 shows that the density of the prepared DES ranged from 1.07 to 1.09 g cm^−3^. The density of DESs tend to be higher than water density and rarely exceeds a value of 2.4 g cm^−3^ (which is the accepted upper limit value for this type of mixture) [14]. The viscosities in the new DESs vary between 159 and 2146 mPa.s (Table 1). These values were measured according to a percentage factor of the measurement made at a given temperature, revolutions per minute (rpm), and spindle size (L_n_) (see Table 1). [DPTAC][Urea] is a white solid at room temperature; it was heated to the minimum temperature at which it becomes a viscous liquid, generating a partial break in the hydrogen bonding network and thus becoming a liquid. Viscosity is of key importance in DESs design, and it is associated with the strength of the interaction between the HBD and the HBA and the molar relationship between the two components of DES [15]. This property can be described according to the ‘hole theory,’ which assumes that, after melting, the ionic material contains empty spaces arising from thermally generated local density fluctuations [16]. The viscosity values obtained in these DESs suggest that their lattice energy is quite significant. This supports the presence of hydrogen bond networks between DES components [7]. Ethylene glycol-based DES ([DPTAC][Eg]) and glycerol-based DES ([DPTAC][Gly]) presented the lowest and the highest viscosities, respectively, which is consistent with previous studies [7]. DES containing lactic acid, urea, or glycerol have strong hydrogen donors that form an important intermolecular hydrogen bonding network with compound **2** and therefore exhibit strong, cohesive energies that arise at high viscous values [14].

Table 2 shows the melting point (mp) for each bio-based DESs and their HBD. As expected, these values are lower in all the eutectic mixtures compared to the HBD. This is attributed to the strength of the interaction between HBD and HBA present in the DESs. This decrease in the melting point is actually due to the combination of hydrogen bonding, bond energy, and entropy changes [17]. Therefore, it is difficult to make predictions about the resulting melting points in these eutectic mixtures [18,19].

### 2.2. Evaluation of Olive Pomace

**Figure 2:**
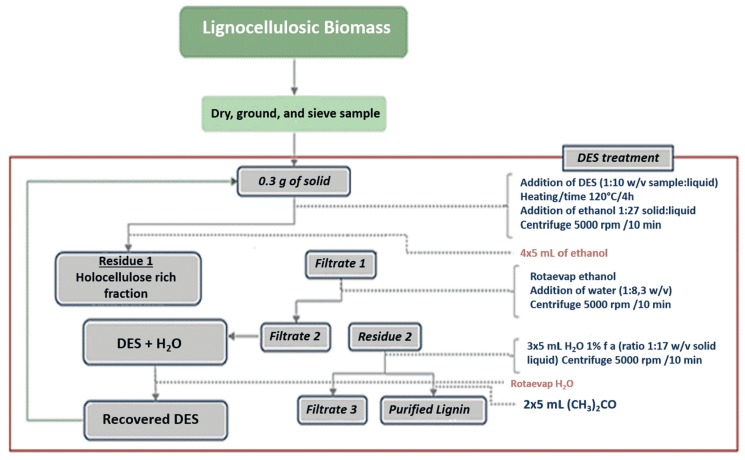
The general procedure of the fractionation of lignocellulosic biomass.

Cellulose, hemicellulose, and lignin are the most common components of biopolymers investigated in biomass [2]. This paper focuses on the fractionation processes of different lignocellulosic biomass (olive pomace and branches from fruit pruning, all hardwood biomass) using DES. Figure 2 shows the multi-step procedure performed for such fractionation.

The results were compared with extractions performed using the DES (ChCl:LA) and results reported by Cequier et al. [21], using IL [Et_3_NH][HSO_4_] (Table 3). The percentage of lignin or holocellulose rich fraction recovered from the DESs was calculated using Equations (1) to (3):

(1) lc= total weight of sample (g)∗% of total lignin or holocellulose in each sample

(2) $$\%\;R=\frac{\mathrm{weight}\;\mathrm{of}\;\mathrm{purified}\;\mathrm{polymer}\;\mathrm{obtained}\;\left(g\right)\;}{\mathrm{lc}\;\left(g\right)}\times 100$$

(3) $$\%\;T=\frac{\mathrm{weight}\;\mathrm{of}\;\mathrm{purified}\;\mathrm{polymer}\;\mathrm{obtained}\;\left(g\right)}{\mathrm{total}\;\mathrm{sample}\;\mathrm{weight}\;\left(g\right)}\times 100$$

where lc = total mass of raw lignin or holocellulose content in each sample. R = Percentage of lignin or holocellulose obtained with respect to lc value. T = Percentage of lignin or holocellulose obtained with respect to the total amount of sample used. (The total content of lignocellulosic material in samples is described in Supplementary Table S3).

Table 3 shows the recovery yields using bio-based DESs. These yields ranged from 23% to 43% (*w*/*w*) of the available lignin in the mg scale (taking into account that the percentage of the total lignin content in dry olive pomace was 37% *w*/*w*). The highest lignin yield was obtained using [DPTAC][LA] for 4 h (43% and 38% *w*/*w* at 120 °C and 150 °C, respectively) followed by [DPTAC][Eg] (34% *w*/*w*). The higher lignin recovery in the extraction of lignocellulosic material using lactic acid-based DES suggests a higher capacity to donate and accept protons by this DES, which can be related to the pH of [DPTAC][LA], the lowest of all bio-based DESs studied (Table 2). This fact improves the chances of hydrogen bonding and thus increases the dissolving ability of DES [22]. A very satisfactory lignin recovery was also obtained by scaling up to 40 g of olive pomace (39% *w*/*w*), as well in the holocellulose recovery (44% *w*/*w*) (Table 3). Thus, lignin and holocellulose recoveries were quite similar to those of the mg scale (38% *w*/*w* and 39% *w*/*w*, respectively), even using a different reactor and stirring system.

The recovery percentages of the non-soluble holocellulose-rich fraction ranged from 25% to 45% (*w*/*w*) (taking into account that the percentage of the total content of cellulose and hemicellulose in the olive pomace was 17% *w*/*w*). The best results in these cases were achieved using the urea-based DES [DPTAC][Urea] (45% *w*/*w*), followed by [DPTAC][LA] for 4 h at 120 °C and [DPTAC][Gly] (39% and 38% *w*/*w*, respectively), and finally [DPTAC][Eg] (34% *w*/*w*). These values are in line with similar studies performed by Kroon et al. [23].

Extraction for 4 h at 120 °C of olive pomace using [DPTAC][LA] (1:1) was also studied to determine if this ratio could improve the yields of the fractionation process [17]. However, recoveries were 32% and 26% for holocellulose-rich fraction and lignin, respectively. These low recoveries led us to finally use the 1:2 ratio in the subsequent experiments.

Olive pomace was also extracted using the IL [Et_3_NH][HSO_4_] following the procedure described in Figure 2. Based on the acidity (Table 1), the IL may have stronger hydrogen bonds than those presented for the bio-based DESs. Therefore, the delignification process using the IL should be higher than that resulting from using DESs due to the lower pH in IL [6], which is consistent with the results indicated in Table 3. However, lignin recoveries considering only DESs showed that the acidity of the DES is important but is not the central parameter for the delignification of the lignocellulosic material. In fact, DES with pH 2–8 produced the same or better lignin recoveries. The most acidic DES was ChCl:LA (pH = 0.9), which gave the worst lignin recovery (9% *w*/*w*) among all the DESs.

On the other hand, the fraction rich in cellulose had two different behaviors. When using bio-based DES, the solid recovered was a mixture of cellulose and hemicellulose (holocellulose), albeit with low yields (7–9% *w*/*w*). However, when using the IL or the ChCl:LA, the residue was enriched in cellulose, which was caused by the dissolution of the hemicellulose and the delignification process.

Although the same treatment was performed on all samples, different behaviors were observed in the amount of lignocellulosic material dissolved in the DES/IL used. The freeze-dried ‘filtrate 3′ fraction (Figure 2) presented a percentage of roughly 70% (*w*/*w*) of the total starting biomass in all-new DESs studied. The FTIR, NMR, Bradford’s method and ash content studies suggested the presence of polyhydroxylated compounds, proteins, some residual DES, and inorganic salts (see Supplementary data).

The eutectic mixture [DPTAC][LA], which had given the highest lignin recovery in olive pomace, was used to perform the fractionation of pruning waste samples in five fruit branches (apricot, plum, peach, nectarine, and flat peach). The extractions were performed at 120 °C and not at 150 °C, due to the lower recovery of the fraction rich in holocellulose and to the similar recovery of lignin and Klason lignin. The percentage of lignin recovered was 57% (*w*/*w*) for apricot, 40% (*w*/*w*) for nectarine, 25% (*w*/*w*) for peach, 20% (*w*/*w*) for flat peach, and 12% (*w*/*w*) for plum (Table 4). It is somewhat surprising that the highest lignin recovery corresponded to the lowest lignin percentage in the pruning waste. However, several factors may play an important role in the delignification process, such as the swelling of the material, the number of carbohydrates linked to lignin, and the ratio between syringyl and guaiacyl units in the lignin structure [14]. Similar results were obtained for a holocellulose-rich fraction of the pruning of these fruit branches using [DPTAC][LA], in which the highest percentage of holocellulose-rich fraction decreased in the order: apricot > peach > nectarine > flat peach > plum (Table 4). Holocellulose yields presented percentages between 10% and 27% (*w*/*w*). In any case, the yields on pruning waste samples of five species of fruit branches with [DPTAC][LA] were not higher than those obtained in the olive pomace sample. Finally, lignin recovered from the different samples using DESs and the IL were treated with 72% sulfuric acid to determine the acid-soluble lignin fraction (Klason lignin) [24]. The percentages corresponding to the Klason lignin are shown in Table 3 and Table 4.

[DPTAC][LA] can be classified as BADES (Brønsted acidic deep eutectic solvent) [25], whose acidity can cause the esterification of hydroxyl groups and the hydrolysis of esters and glycosidic bonds [26]. Hydrolytic processes need the presence of water, whereas the esterification process could be favored at high temperatures [26]. The dissolution of cellulose and hemicelluloses in this DES could be promoted for the hydrolytic processes, although in our experiments, water was only present in a small percentage since dry materials were used and the DES used had a low moisture content. Nevertheless, some hydrolytic processes cannot be discarded, which could explain the higher lignin recovery and the slightly lower holocellulose recovery when [DPTAC][LA] was used instead of the other DESs. The esterification process could also be the cause of a higher yield on the holocellulose and lignin recoveries due to the increase in molecular weight when esterification occurs. Esterification processes would explain the increase of the band around 1735 cm^−1^ in some FT-IR spectra. The increase of this band is easily observed in the nectarine material and at 150 °C with the olive pomace, which would indicate different structural features among the different hardwood materials used (see Supplementary Material, bands at 1735 cm^−1^ and 1737 cm^−1^ in Figures S20 and S22, respectively). However, this increase cannot be observed using [DPTAC][LA] at 120 °C when olive pomace and the other pruning material were used (see Supplementary Material Figures S17–S19, S21 and, S23).

### 2.3. Instrumental Characterization

#### 2.3.1. Gel Permeation Chromatography (GPC)

The molecular weight of the extracted lignin was determined by GPC (see Supplementary Material Table S4). Surprisingly, the average molecular weight (Mw) from olive pomace and the pruning wastes fell beyond the upper limit of our system, and therefore these determinations must be regarded as qualitative. Nevertheless, for the comparative purposes of this study, the data acquired are sufficient to draw relevant conclusions. In general, [DPTAC][LA] provided polymers with much lower Mw than using IL. In the olive pomace samples, the Mw of the lignin extracted with [DPTAC][LA] was 5.5 times lower than using the IL. Apricot pruning resulted in the lignin with the lowest Mw among all the pruning species studied. The polydispersity values (PDI) of the lignin obtained with [DPTAC][LA] (2.3–2.6) were also significantly lower than those obtained with the IL (6.3–9.6). In light of these results, the best approach to obtain lignin with the lowest Mw and PDI and with high yields is to use [DPTAC][LA].

It is well known that lignin undergoes, to some extent, processes of re-polymerization and condensation when extracted. These processes change the chemical structure of the lignin and, therefore, its molecular weight. For example, Tan et al. [27] reported that lignin extracted with the ionic liquid [C_2_C_1_im][ABS] had a lower molecular weight than the lignin obtained by aqueous auto-catalyzed pre-treatment. In our case, the ionic liquid has a pH of 0.5, while the pH of [DPTAC][LA] is higher (pH = 1.2). Our group has already published the differences between three different extractions of lignin from olive pomace using an aforementioned IL, alkaline (pH = 13 with NaOH), and acid treatments (H_2_SO_4_, 72%) [21]. The lowest molecular weight observed corresponded to the lignin extracted using the ionic liquid, which was approximately 40% lower than those resulting from the other two methods. It is therefore not surprising that the molecular weight of the extracted lignin differs according to whether the IL or DES were used (results are shown in the Supplementary Material Table S4).

#### 2.3.2. FT-IR Spectra

**Figure 3:**
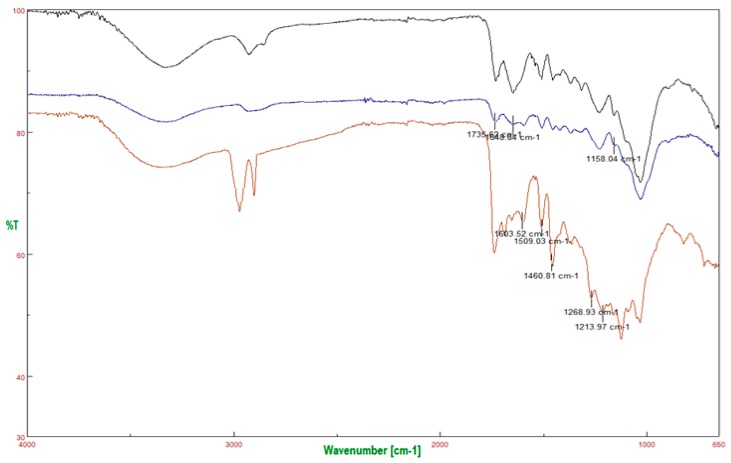
FT-IR spectra of olive pomace before treatment (black line), holocellulose-rich fraction (blue line), and extracted lignin (red line) using [DPTAC][LA].

Fourier-transformed infrared spectrometry was used to characterize the chemical structure of the lignocellulosic biomass. The FT-IR spectra of lignin and holocellulose-rich fraction from olive pomace extracted using [DPTAC][LA] are shown in Figure 3, as well as their FT-IR signals from characteristic functional groups (summarized in Table 5). FT-IR spectroscopy indicates a high delignification of the holocellulose-rich fraction obtained by treatment with all DESs (see Supplementary Material).

According to the FT-IR spectra, differences between lignin (Figure 3, black line) and the holocellulose-rich fraction (Figure 3, blue line) were found in the 1657–1720 cm^−1^ fingerprint region, where overlapping bands corresponding to carbohydrates and lignin structure were found. The group of bands found in this region showed vibrations corresponding to -O-CH_3_, C-O-C, and C=C groups, which probably indicates their high content in the lignin structure [28]. The bands at 1737–1751 cm^−1^ drastically decrease in intensity after washing with acetone, indicating that these bands corresponded to fatty material present in the samples. The absence of bands at 1705–1715 cm^−1^ and 1650 cm^−1^ indicated that no unconjugated carbonyl-carbonyl vibrations were detected. Concerning the FT-IR spectra of the holocellulose-rich fraction, the band at 2925 cm^−1^ is attributed to the C–H vibration within the methylene of the holocellulose. The group of broad bands located at 1200–1000 cm^−1^ are related to the structure of the cellulose. However, these bands could overlap the C–O–H vibrations bands of primary and secondary alcohols at 1061 cm^−1^, C–O–C glycosidic bond at 1161 cm^−1^ and C–O–C ring skeletal vibration at 1107 cm^−1^. On the other hand, the 1510 and 1490 cm^−1^ bands were used to follow the delignification process in all samples. Although this fraction is mostly formed by cellulose, the presence of a small lignin-associated band at 1516 cm^−1^ that corresponds to aromatic skeletal vibrations was also observed. The bands at 2854 and 1456 cm^−1^ are produced by C-H deformation within the lignin methoxyl groups [29,30].

Finally, infrared studies of the fractions resulting from the pruning of five fruit trees (see Supplementary Material) show that the lignin extractions followed the same behavior than those carried on olive pomace (all of them using [DPTAC][LA]).

#### 2.3.3. H NMR Spectra

The ^1^H NMR spectrum of the lignin fraction extracted from olive pomace using [DPTAC][LA] showed many important structural features of lignin including the signals of β–5 phenolic hydroxyl (δ 8.99 ppm), phenolic hydroxyl (δ 8.1–9.4 ppm), syringyl C-5 phenolic hydroxyl (δ 8.1–8.5 ppm). Moreover, signals between δ 5.3 and δ 7.5 ppm were assigned to aromatic protons of guaiacyl units (G). The signals at δ 3.35–4.06 ppm can be assigned to protons of methoxyl (-OCH_3_) groups. The strong signal at 3.35 ppm is closely linked to the guaiacyl units, whereas those between δ 0.5 and 1.0 ppm corresponded to aliphatic protons. (for the full ^1^H NMR spectra of the samples studied see Supplementary Material). These results are in full agreement with the study performed on Kraft lignin by Diop et al. [31].

## 3. Materials and Methods

### 3.1. Materials

Acetone (99%) and deuterated solvents for NMR analysis (chloroform-*d* 99.8 atom% D, DMSO-*d*_6_ 99.9 atom% D, methanol-*d*_4_ 99.8 atom% D, acetone-*d*_6_ 99.9 atom% D) were purchased from Sigma–Aldrich (St. Louis, MO, USA); Benzoic acid (≥99.5%) was purchased from Fluka (Honeywell, Charlotte, USA). Glycerol (≥99%), DL-lactic acid (90%), ethylene glycol (technical grade), and triethylamine (99%) were purchased from Acros (Thermo Fischer Scientific, Waltham, MA, USA). Ethanol (96%) and methanol (96%) were purchased from Scharlau (Barcelona, Spain). Hydrochloric acid (38%) was purchased from Baker (Phillipsburg, NJ, USA). Urea (≥99%) was purchased from Panreac (Barcelona, Spain), and glacial acetic acid (≥99.5%) was purchased from Labkem (Dublin, Ireland).

Animal fat was kindly donated by ‘Subproductos Cárnicos Echevarria y Asociados S.L’ (Cervera, Spain). Pruning waste of fruits trees (apricot, peach, nectarine, flat peach, and plum) was provided by ‘Grupo Catalá’ (La Portella, Spain), the total content of lignin and cellulose was determined in all samples: apricot: 14% lignin, 37% cellulose; plum: 26% lignin, 41% cellulose; peach: 18% lignin, 40% cellulose; nectarine: 16% lignin, 41% cellulose; and flat peach: 28% lignin, 36% cellulose. Samples of olive pomace were provided by ‘Agrícola de l’Albi’ (L’Albi, Spain); they had a total content of 37% lignin, 17% cellulose. Pruning waste material and olive pomace were dried, ground, sieved (1 mm particle size), and stored in a desiccator before extraction. In this study, the amount of 0.3 g was used for the preliminary experiments and 40 g for the scale-up of the extraction. All lignocellulosic material was extracted, applying the same protocol indicated by Cequier et al. [21]. Samples were extracted with DESs in a ratio solid/liquid 1:10 or 1:5 (*w*/*v*) from 100 °C to 150 °C for 2 h and 4 h. Magnetic stirring at 650–670 rpm and mechanical stirring at 500 rpm were used to extract 0.3 g and 40 g samples, respectively. Ethanol was added in a ratio 1:27 *w*/*v*, and the mixture centrifuged at 5000 rpm for 10 min. The pellet was recovered by filtration and was washed with ethanol (4 × 5 mL). The filtrate was concentrated under vacuum and water was added in a ratio 1:8 (*w*/*v*). The mixture was centrifuged at 5000 rpm for 10 min to obtain a solid precipitate. This precipitate was washed with H_2_O: formic acid 1% in a ratio 1:17 (*w*/*v*) and centrifuged at 5000 rpm (3780 RCF) for 10 min. The final product was washed with acetone to remove fatty acids when present.

### 3.2. Solvent Evaporations

Solvents were removed under reduced pressure in a Büchi Rotavapor R-210 (Flawil, Switzerland). Centrifugations were performed in a Hettich Zentrifugen EBA21 GmbH & Co. (3780 RCF/10 min) (Tuttlingen, Germany).

### 3.3. Viscosity

Viscosity values were determined using a J.P. Selecta rotational viscometer ST-2001-L (Barcelona, Spain) and measured in a 250 mL beaker, where 175 mL of each DES was placed. Values were recorded between 60 and 100 rpm, temperature between 80 °C to 94 °C and the appropriate size of the rotational spindle (L_1_, L_2_, and L_3_) selected according to the viscosity of the sample.

### 3.4. Melting Points

Melting points could not be measured according to standard protocols because DESs are viscous liquids except [DPTAC][Urea] (described below), which was measured in a ‘Gallenkamp’ melting point apparatus. Measures were obtained by freezing 250 mL of each sample at –80 °C, and then allow them to warm until they had melted, monitoring the process with a thermometer (–50 °C to 30 °C scale).

### 3.5. NMR Spectra

Spectra were recorded on a Varian NMR spectrometer (400 MHz, δ: ppm), (Palo Alto, CA, USA).

### 3.6. FT-IR Spectra

Spectra were performed on a Jasco FT-IR 6300 spectrometer (Tokyo, Japan), in a range of 4000-650 cm^−1^.

### 3.7. Mass Spectrum

Mass spectrum was recorded with a UPLC (Acquity)-MS/MS (Xevo TQS), (Santa Clara, CA, USA). The analysis was carried out by direct injection and combining the sample with mobile phase (MeOH + H_2_O (90 + 10 *v*/*v* 0.1% formic acid) with an Electrospray probe (ESI), Source temperature = 150 °C, Desolvation temperature = 400 °C.

### 3.8. Gel Permeation Chromatography (GPC)

Experiments were carried out on a Varian ProStar instrument equipped with a UV-vis detector (260 nm) and two PolarGel-L columns (300 × 7.5 mm) at 50 °C. The mobile phase consisted of DMSO with 0.1% lithium bromide, the flow rate was 0.8 mL/min. Calibration of the system ranged from 162 to 19,500 g/mol with polystyrene standards (Sigma–Aldrich).

### 3.9. Synthesis of 3-Chloro-1,2-propanediol **1**

The starting glycerol was obtained from animal fat, according to Gallart et al. [32] and purified prior used [33]. A mixture of 300 g of this glycerol, 600 mL of hydrochloric acid, and 15 g of glacial acetic in a 2000 mL flask was heated under reflux for 10 h. As the reaction progressed and the evolution of acid vapors diminished, the mixture was heated more intensely [34]. The desired product was recovered by distillation at 115–117 °C/11 mmHg (63% yield). ^1^H NMR: (400 MHz, CDCl_3_, δ: ppm) = 3.90 (2H, d, *J* = 5.6, CH_2_OH), 3.73 (1H, m, CHOH), 3.60, 3.52 (2H, m, CH_2_Cl). ^13^C NMR: (400 MHz, CDCl_3_, δ: ppm) = 45.72 (CH_2_-Cl), 63.74 (H_2_C-OH), 71.90 (H-C-OH).

### 3.10. Synthesis of [C_9_H_22_N^+^O_2_]Cl^−^ **2**

Synthesis was adapted from the procedure reported by Beckett et al. [35]. A 4.2 M solution of ethanolic triethylamine (100 mmol) was cooled on an ice bath, then 3-chloro-1,2-propanediol (100 mmol) was added slowly using a syringe, followed by methanol (60 mL). The mixture was heated under reflux (60 °C) overnight, and the solvent was removed by rotary evaporation to yield a crude yellow oil. Small portions of the oil were washed with a large excess of acetone to afford a white hygroscopic powder (56% yield). Compound **2** was characterized by NMR and FT-IR techniques. ^1^H NMR: (400 MHz, DMSO *d*_6_, δ: ppm) = 1.18 t (9H), 3.03 q (1H), 3.25 dd (1H), 3.39 m (3H), 3.48 dd (3H), 3.56 m (1H), 3.63 q (1H), 3.96 m (1H), 5.28 t (OH), 5,74 d (OH). ^13^C NMR: (400 MHz, DMSO d6, δ: ppm) = 7.3 (CH_3_), 52.98 (CH_3_CH_2_-N), 59.54 (CH_2_-N)a, 62.59 (CH_2_-N)b, 63.58 (CH_2_-N)c, 65.45 (CH-OH), 71.29 (CH_2_-OH). FT-IR (ν Max/cm^−1^) = 3284.18 (OH), 3216.68 (OH), 2986.23, 2924.52 (C-H alkyl groups), 2885.92, 2817.49 (N^+^CH), 1489.74 ((CH_2_)^3^-N^+^), 1398.14 (CH_2_-N), 1373.07, 1292.07, 1163.83, 1087.66 (C-N), 1150.83 (C-O-C), 1040.00 (CO),1002.8 ((CH_2_)_3_-N^+^), 961.341, 937.235, (C-C), 847.56 (CH), 793.564 (γ CH_2_), 703.89 (CH_2_). *m*/*z* obs: 176.19 (C_9_H_22_N^+^O_2_); 158.02 (C_9_H_22_N^+^O_2_ − H_2_O).

### 3.11. Synthesis of DESs

The preparation of novel DESs (Figure 1) was based on the procedure reported by Abbott et al. [36]. The eutectic mixtures were prepared by stirring compound **2** at 80 °C with either lactic acid, urea, commercial glycerol, or ethylene glycol in a 1:2 or 1:1 stoichiometric ratio until a homogeneous colorless liquid was formed. In addition, the preparation of two other potential DESs using compound **2** and either citric acid and benzoic acid was intended. Nevertheless, the eutectic mixture between compound **2** and citric acid decomposed at around 80 °C, whereas no eutectic mixture was achieved using benzoic acid.

[DPTAC][LA]: ^1^H NMR (400 MHz, DMSO *d*_6_, δ: ppm) = 1.26 m (12H), 3.34 m (10H), 4.02 m (2H), 4,18 m (1H), 4.91 m (1H), 5.23 bs (OH), 5.65 bs (OH). FT-IR (ν Max/cm^−1^) = 3331.18 (OH), 2986.23, 1456.96, 1372.1 (CH_3_), 1729.83 (C=O), 1203.36, 1124.3, 1042.34 (C-O).

[DPTAC][Urea]: ^1^H NMR (400 MHz, DMSO *d*_6_, δ: ppm) = 1.20 t (9H), 3.34 m (18H), 3.95 dd (1H), 5.21 t (1H), 5.51 bs (4H), 5.61 d (OH), 5.93 d (OH). FT-IR (ν Max/cm^−1^) = 3426.89, 3326.61 (N-H), 3255.25, 1154.19 (N-H_2_), 1672.95 (C=O), 1457.92, 1089.58, 1001.84 (C-N), 786.81 (H_2_N-CO).

[DPTAC][Gly]: ^1^H NMR (400 MHz, DMSO *d*_6_, δ: ppm) = 1.16 t (9H), 3.32 m (22 H), 3.97 m (1H), 4.44 t (3H), 4.51 d (1H), 5.19 dd (OH), 5.59 d (OH). FT-IR (ν Max/cm^−1^) = 3296.71 (OH), 2933.2 (CH_2_), 2877.21 (N^+^-CH), 1456.96 (CH_2_), 1395.25 (N-CH_3_), 1337.39 (CH_2_), 1159.97, 1092.48 (C-N), 1110.8, 1035.59 (C-O), 1000.87 ((CH_2_)_3_-N^+^), 928.55 (C-OH), 845.63 (-O-C_2_H_4_).

[DPTAC][Eg]: ^1^H NMR (400 MHz, DMSO *d*_6_, δ: ppm) = 1.18 t (9H), 3.29 m (22H), 3.64 m (1H), 3.97 m (1H), 4.51 t (4H), 4.77 t (1H), 5.21 t (OH), 5.61 d (OH). FT-IR (ν Max/cm^−1^) = 3295.75 (OH), 2938.98, 2873.42 (CH), 1456.96 (CH_2_), 1395.25 (N-CH_3_), 1256.4 (CH_2_), 1159.97 (C-N), 1085.73 (-C-O), 1035.59 (O-C-C-O), 882.27 (CH_2_), 861.06 (C-C).

## 4. Conclusions

Four novel bio-based eutectic solvents have been prepared by mixing proper hydrogen bond acceptors and donors (1:2). The hydrogen bond acceptor was prepared from raw glycerol, while the hydrogen bond donors were commercial products of natural origin. These DESs were used to fractionate lignocellulosic biomass from olive pomace and pruning of several fruit branches. The results show that our DESs were more effective than DES:ChCl:LA to fractionate the lignocellulosic material used (using mainly [DPTAC][LA]). FT-IR and NMR were used to characterize the fractions of the different lignocellulosic constituents. These spectra demonstrated that there were no noticeable changes in the original structure of lignin and holocellulose after treatment with the eutectic mixtures. GPC was used to determine the molecular weight (Mw) and the polydispersity index (PDI) of the lignin isolated. To the best of our knowledge, this is the first report about the preparation of these DESs as well as their application as solvents in the fractionation of the biomass from olive pomace and fruit branches. The use of these DESs as medium in other applications is under study. The usefulness of the isolated holocellulose and lignin is also underway. Both studies will be reported in due course.

## Figures and Tables

**Table 1:** Physicochemical properties of DESs and the IL used in this study.

DES and IL	δ [g.cm^−3^]	η ^a^ [mPa.s]	Apparentp H
[DPTAC][LA]	1.07	1357	1.20
[DPTAC][Urea]	1.09	383	7.78
[DPTAC][Gly]	1.07	2146	2.39
[DPTAC][Eg]	1.08	159	1.15
[Et_3_NH][HSO_4_] ^b^	1.18	81	0.5
ChCl:LA ^c^	1.16	58	0.9

Note: ^a^ Conditions for [DPTAC][LA] = 100 rpm, 80.7%, 25 °C, L_3_; [DPTAC][Urea] = 60 rpm, 91.6%, 78 °C, L_3_; [DPTAC][Gly] = 100 rpm, 76%, 25 °C, L_2_; and [DPTAC][Eg] = 50 rpm, 93.3%, 25 °C, L_1._^b^ IL contained 20% of water, 100 rpm, 93.8%, 25 °C, L_1_. ^C^ ChCl: LA (1:10 molar ratio), 100 rpm, 68.2%, 25 °C, L_1_. pH value of DESs precursor **2** at 85 °C was 1.10.

**Table 2:** Melting points (mp) for the HBDs components of the DESs (the pKa of HBD is also provided).

HBD	pKa	mp [°C]	DES	mp [°C]
Lactic acid (LA)	3.86 ^a^	18	[DPTAC][LA]	−32
Urea	14.4^b^	133	[DPTAC][Urea]	65–75
Glycerol (Gly)	14.4 ^a^	18	[DPTAC][Gly]	−28
Ethylene glycol (Eg)	15.1 ^a^	−13	[DPTAC][Eg])	<−56

**Note:**^a.^ pKa extracted from https://pubchem.ncbi.nlm.nih.gov (accessed April 2020); ^b.^ pKa extracted from Makarov et al. [20]. The melting point value of compound **2** is 96 °C.

**Table 3:** Results of the lignin extraction and holocellulose recovery from olive pomace using DESs and IL.

	Holocellulose-Rich Fraction			Lignin			
DESs and IL	Weight [mg]	Recovery ^a^ [%]	Total ^b^ [%]	Weight [mg]	Recovery ^a^ [%]	Total ^b^ [%]	Klason ^e^ [%]
[DPTAC][LA]	23	39	8	42	38	14	60
[DPTAC][LA] (g scale)	3.02 × 10^3^	44	7.6	5.6 × 10^3^	39	14	59
[DPTAC][LA] ^g^	13	25	4.3	29	27	10	56
[DPTAC][LA] ^h^	15	29	5	34	30	12	52
[DPTAC][LA] ^i^	18	27	6	9	8	3	
[DPTAC][LA] ^j^	19	32	6.5	47	43	16	63
[DPTAC][Urea]	27	45	9	30	27	10	57
[DPTAC][Gly]	23	38	8	30	27	10	57
[DPTAC][Eg]	20	34	7	37	34	12	58
	**Cellulose Rich Fraction**						
[Et_3_NH][HSO_4_] ^c^	159	N/A ^d^	53	67	60	22	98 ^f^
ChCl:LA	239	N/A ^d^	76	26	23	9	--

Note: **^a^** % of lignin and holocellulose-rich fraction calculated from the available lignin cellulose and hemicellulose in samples; **^b^** % of lignin and cellulose or holocellulose-rich fraction calculated from the total weight; **^c^** Mean value (n = 6; coefficient of variation = 31%); **^d^** Not applicable (N/A) since recoveries were >100% because cellulose-rich fractions contained other components. **^e^** % values with respect to **^a^** (recovery values). **^f^** percentage determined by considering the total available lignin in the raw biomass. **^g^** Extraction performed with DESs in a ratio solid/liquid (1:5) for 4 h. **^h^** Extraction time performed for 2 h at 120 °C. **^i^** Extraction performed at 100 °C for 4 h. **^j^** Extraction performed at 150 °C for 4 h.

**Table 4:** Results from lignin extraction and holocellulose recovery using [DPTAC][LA] in pruning waste of five different fruit branches.

[DPTAC][LA]	Holocellulose-Rich Fraction			Lignin			
Sample	Weight [mg]	Recovery [%]	Total [%]	Weight [mg]	Recovery [%]	Total [%]	Klason [%]
Apricot	52	27	17	24	57	8	45
Plum	15	10	5	9	12	3	48
Peach	34	23	11	13	25	5	48
Nectarine	27	18	9	19	40	7	45
Flat peach	26	19	9	16	20	6	46

**Table 5:** Wavenumber assignments of the characteristics of IR bands of lignin (**A**) and holocellulose-rich fraction (**B**).

A) IR Bands Assignments for Lignin		B) IR bands Assignments for the Holocellulose Rich Fraction	
Wavenumber (cm^−1^)	Band Assignments	Wavenumber (cm^−1^)	Band Assignments
3600–3000	ν OH aromatic and aliphatic	3350	ν OH
2960–2925	ν CH_3_-CH_2_	2925	ν methylene and methyl groups
2921	ν methyl and methylene	2800	ν CH_2_ stretch
2860, 1460	ν and deform CH.	1642	H_2_O
1720	C=O fatty acid band	1605	ν cellulose-H_2_O
1657	ν C=O carbonyl-carboxyl	1430	CH_2_ def.
1639	ν C=O alkyl group	1368	C-H def.
1610	ν aromatic	1200–1000	ν typical bands cellulose
1516	ν aromatic	1161	ν C-O-C glucosidic
1597	ν aromatic	1107	ν C-O-C ring
1506	ν aromatic	1033	ν cellulose and hemicell. (broad band)
1427	CH def.	1058,1159, 1157	ν C-O-C pyranose ring
1425	ν aromatic ring	910	β(1-4) C-O-C
1375, 1330	ν OH aromatics	895	β-glucosidic
1364	ν CH		
1370	ν Syringyl groups		
1264	ν Guaiacyl groups		
1200	OH carbohydrates		
1120	ν Syringyl groups		
1111	ν glucose ring		
825	ν Syringyl group		
916, 810	ν guaiacyl group		

## Supplementary Materials

The Supplementary Materials are available online. **Figure S1.** ^1^H NMR of 2. **Figure S2.** FT-IR spectra of **2**. **Figure S3. a**) FT-IR spectra of lactic acid. **Figure S3. b**) FT-IR spectra of [DPTAC][LA]. **Figure S4. a**) FT-IR spectra of urea. **Figure S4. b**) FT-IR spectra [DPTAC][Urea]. **Figure S5. a**) FT-IR spectra of glycerol. **Figure S5. b**) FT-IR spectra of [DPTAC][Gly]. **Figure S6. a**) FT-IR spectra of ethylene glycol. **Figure S6. b**) FT-IR spectra of [DPTAC][Eg]. **Figure S7. a**) ^1^H NMR spectra of lactic acid. **Figure S7. b**) ^1^H NMR spectra of [DPTAC][LA]. **Figure S8. a**) ^1^H NMR spectra of urea. **Figure S8. b**) ^1^H NMR spectra of [DPTAC][Urea]. **Figure S9.** ^1^H NMR and FT-IR of naturally obtained glycerol. **Figure S10. a**) ^1^H NMR spectra of glycerol. **Figure S10. b**) ^1^H NMR spectra of [DPTAC][Gly]. **Figure S11. a**) ^1^H NMR spectra of ethylene glycol. **Figure S11.b**) ^1^H NMR spectra of [DPTAC][Eg]. **Figure S12**. Pictures for all DESs. **Figure S13**. FT-IR spectra of ‘filtrate 2′ from olive pomace in [DPTAC][LA]. **Figure S1.** ^1^H NMR spectra of filtrate 3 fraction from olive pomace using [DPTAC][LA]. **Figure S15.** FT-IR spectra of filtrate 3 fraction from olive pomace using [DPTAC][LA]. **Figure S16.** FT-IR spectra of ashes obtained from filtrate 3 fraction of olive pomace using [DPTAC][LA]. **Figure S17**. FT-IR spectra of lignin and holocellulose-rich fraction extracted from pruning of apricot branches using [DPTAC][LA]. **Figure S18.** FT-IR spectra of lignin and holocellulose-rich fraction extracted from pruning of plum branches using [DPTAC][LA]. **Figure S19.** FT-IR spectra of lignin and holocellulose-rich fraction extracted from pruning of peach branches using [DPTAC][LA]. **Figure S20.** FT-IR spectra of lignin and holocellulose-rich fraction extracted from pruning of nectarine branches using [DPTAC][LA]. **Figure S21.** FT-IR spectra of lignin and holocellulose-rich fraction extracted from pruning of flat peach using [DPTAC][LA]. **Figure S22.** FT-IR spectra of holocellulose and lignin obtained at 150 °C from olive pomace. **Figure S23.** ^1^H NMR spectra of lignin fraction obtained from olive pomace using [DPTAC][Urea]. **Figure S24.** ^1^H NMR spectra of lignin fraction obtained from olive pomace using [DPTAC][Gly]. **Figure S25.** ^1^H NMR spectra of lignin fraction obtained from olive pomace using [DPTAC][Eg]. **Figure S26.** ^1^H NMR spectra of lignin from olive pomace in [DPTAC][Eg]. **Table S1.** Peak assignments of ^1^H-NMR spectrum of **2**. **Table S2**. Band assignments of FT-IR spectrum of **2**. **Table S3.** Total content of lignocellulosic material. **Table S4.** Molecular weight (Mw) and polydispersity index (PDI) of lignin in samples measured by GPC.
