# Supplementary material for: Effect of Four Novel Bio-Based DES (Deep Eutectic Solvents) on Hardwood Fractionation
Source: Molecules. 2020 May 5;25(9):2157. doi: 10.3390/molecules25092157 (PMC7248933; doi:10.3390/molecules25092157)
Supplement: Supplementary file 1 [file molecules-25-02157-s001.pdf]

SUPPLEMENTARY MATERIAL

Effect of four novel bio-based DES (Deep Eutectic Solvents) on hardwood fractionation

Paulo Torres, Mercé Balcells, Enrique Cequier, Ramon Canela-Garayoa*

University of Lleida, ETSEA.
Department of Chemistry-DBA center.
Av. Rovira Roure 191, 25198, Lleida, Spain.
*Corresponding author(s). Email: canela@quimica.udl.cat
Tel. +34 973702843.

Content **Page**

Figure S1: ^{1}H NMR of **2** 2
Figure S2: FT-IR spectra of **2** 2
Figure S3: a) FT-IR spectra of lactic acid 3
Figure S3: b) FT-IR spectra of [DPTAC][LA] 3
Figure S4: a) FT-IR spectra of urea 4 4
Figure S4: b) FT-IR spectra [DPTAC][Urea] 4
Figure S5: a) FT-IR spectra of glycerol 5
Figure S5: b) FT-IR spectra of [DPTAC][Gly] 5
Figure S6: a) FT-IR spectra of ethylene glycol 6 6
Figure S6: b) FT-IR spectra of [DPTAC][Eg] 6
Figure S7: a) ^{1}H NMR spectra of lactic acid 7
Figure S7: b) ^{1}H NMR spectra of [DPTAC][LA] 7
Figure S8: a) ^{1}H NMR spectra of urea 8
Figure S8: b) ^{1}H NMR spectra of [DPTAC][Urea] 8
Figure S9: ^{1}H NMR and FT-IR of naturally obtained glycerol 9
Figure S10: a) ^{1}H NMR spectra of glycerol 10
Figure S10: b) ^{1}H NMR spectra of [DPTAC][Gly] 10
Figure S11: a) ^{1}H NMR spectra of ethylene glycol 11
Figure S11: b) ^{1}H NMR spectra of [DPTAC][Eg] 11
Figure S12: DESs 12
Figure S13: FT-IR spectra of “filtrate 2” from olive pomace in [DPTAC][LA] 14
Figure S14: ^{1}H NMR spectra of “filtrate 3” from olive pomace in [DPTAC][LA] 14
Figure S15: FT-IR spectra of “filtrate 3” from olive pomace in [DPTAC][LA] 15
Figure S16: FT-IR spectra of ashes from “filtrate 3” of olive pomace in [DPTAC][LA] 15
Figure S17: FT-IR spectra of pruning apricot branches 16
Figure S18: FT-IR spectra of pruning plum branches 16
Figure S19: FT-IR spectra of pruning peach branches 17
Figure S20: FT-IR spectra of pruning nectarine branches 17
Figure S21: FT-IR spectra of pruning flat peach branches 18
Figure S22: FT-IR spectra of holocellulose and lignin obtained at 150°C 18
Figure S23: ^{1}H NMR spectra of lignin from olive pomace in [DPTAC][LA] 19
Figure S24: ^{1}H NMR spectra of lignin from olive pomace in [DPTAC][Urea] 19
Figure S25: ^{1}H NMR spectra of lignin from olive pomace in [DPTAC][Gly] 20
Figure S26: ^{1}H NMR spectra of lignin from olive pomace in [DPTAC][Eg] 20
Table S1: Peak assignments of ^{1}H-NMR spectrum of 2 12
Table S2: Band assignments of FT-IR spectrum of 2 13
Table S3: Total content of lignocellulosic material 13
Table S4: Molecular weight of lignin in samples used by GPC 21

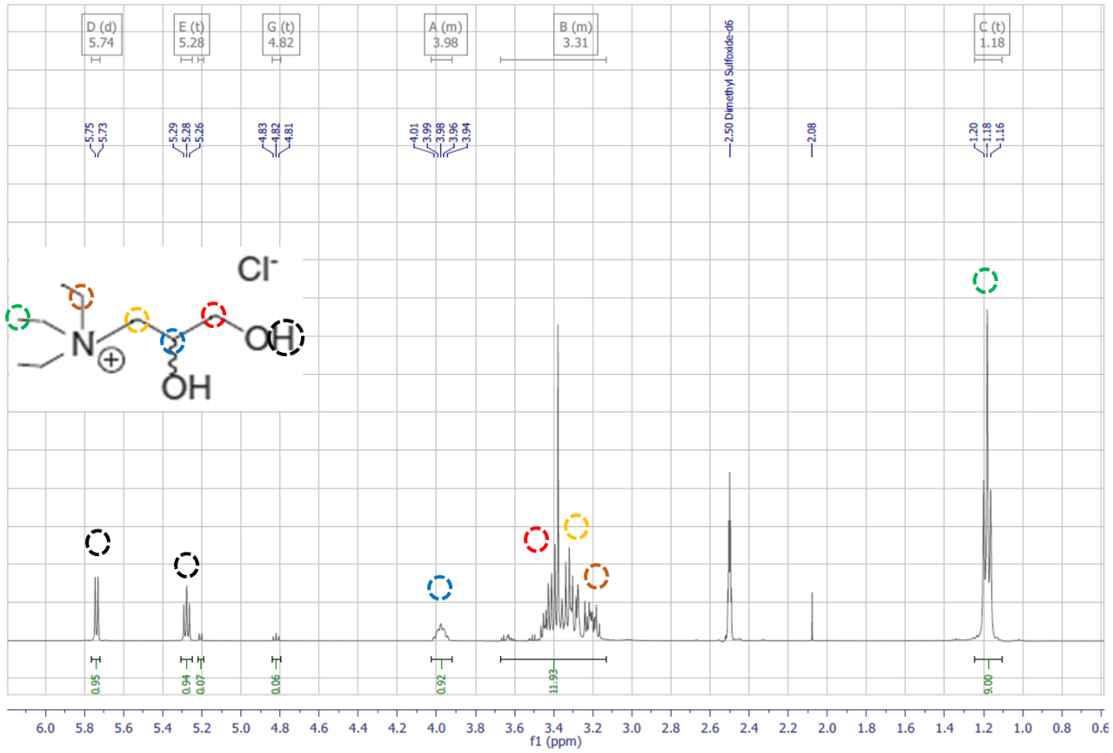

Figure S1: ^{1}H NMR (Dmso d_6, 400 MHz) of precursor **2.**

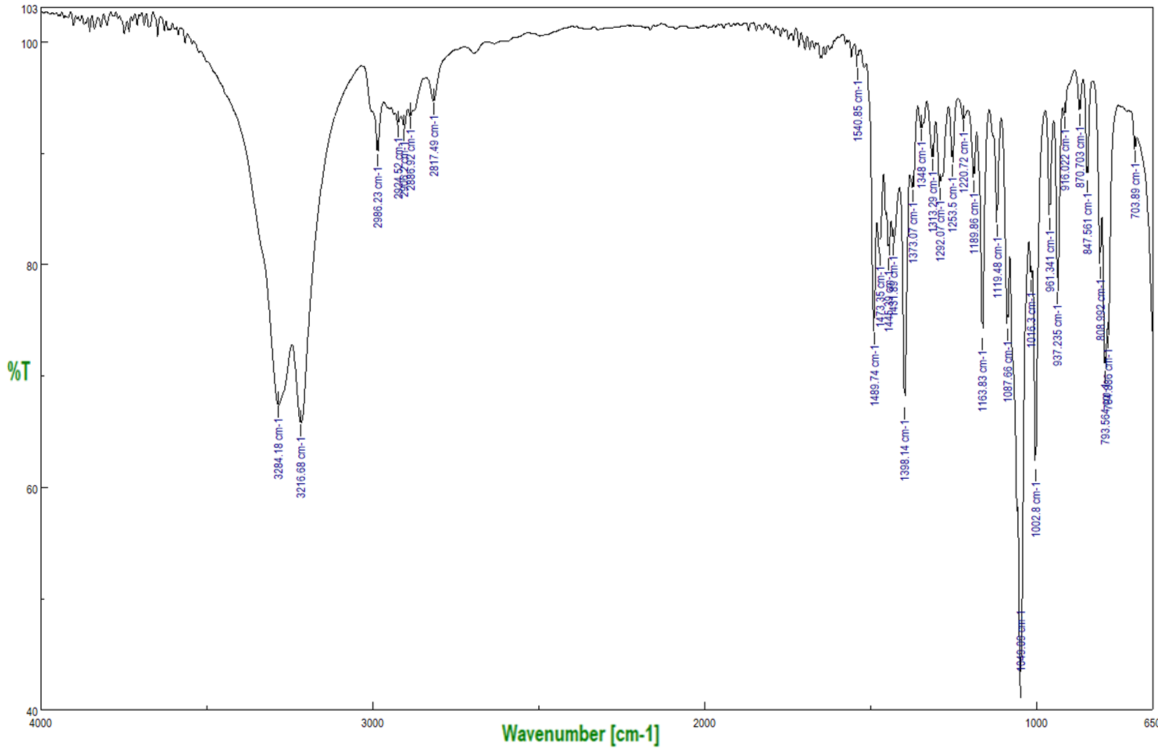

Figure S2: FT-IR spectra of precursor **2.**

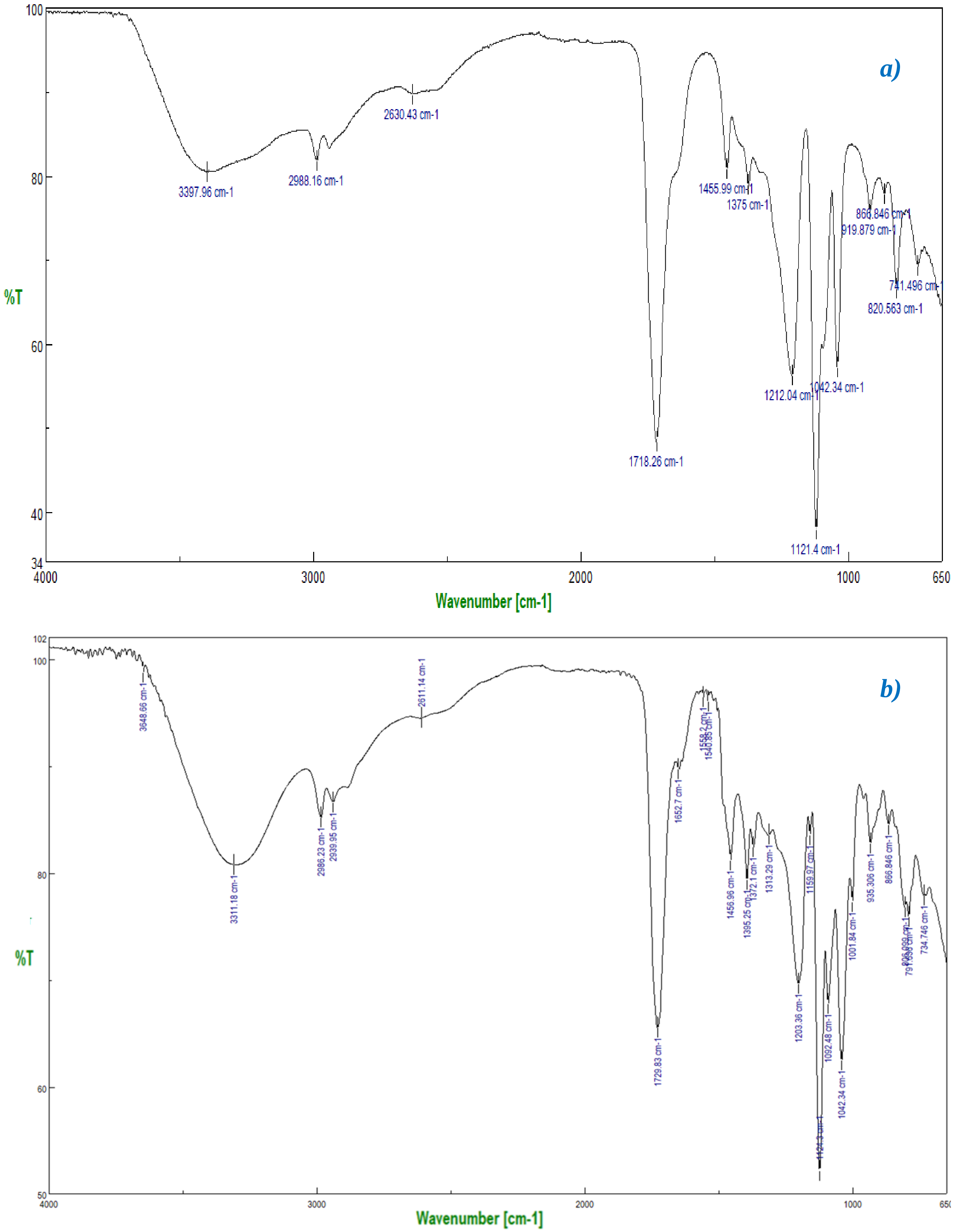

Figure S3: a) FT-IR spectra of lactic acid. **b)** FT-IR spectra of [DPTAC][LA].

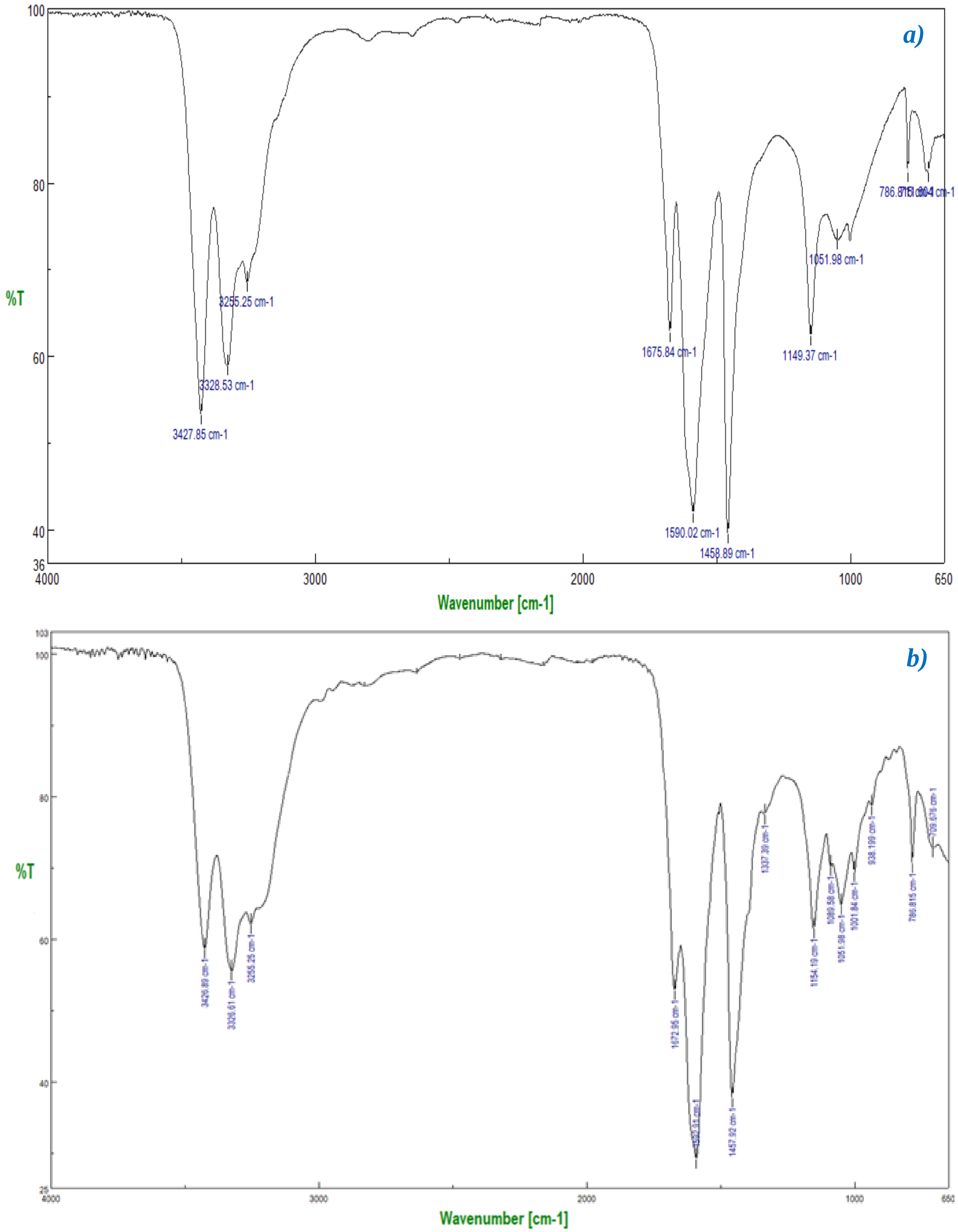

Figure S4: a) FT-IR spectra of urea. **b)** FT-IR spectra of [DPTAC][UREA].

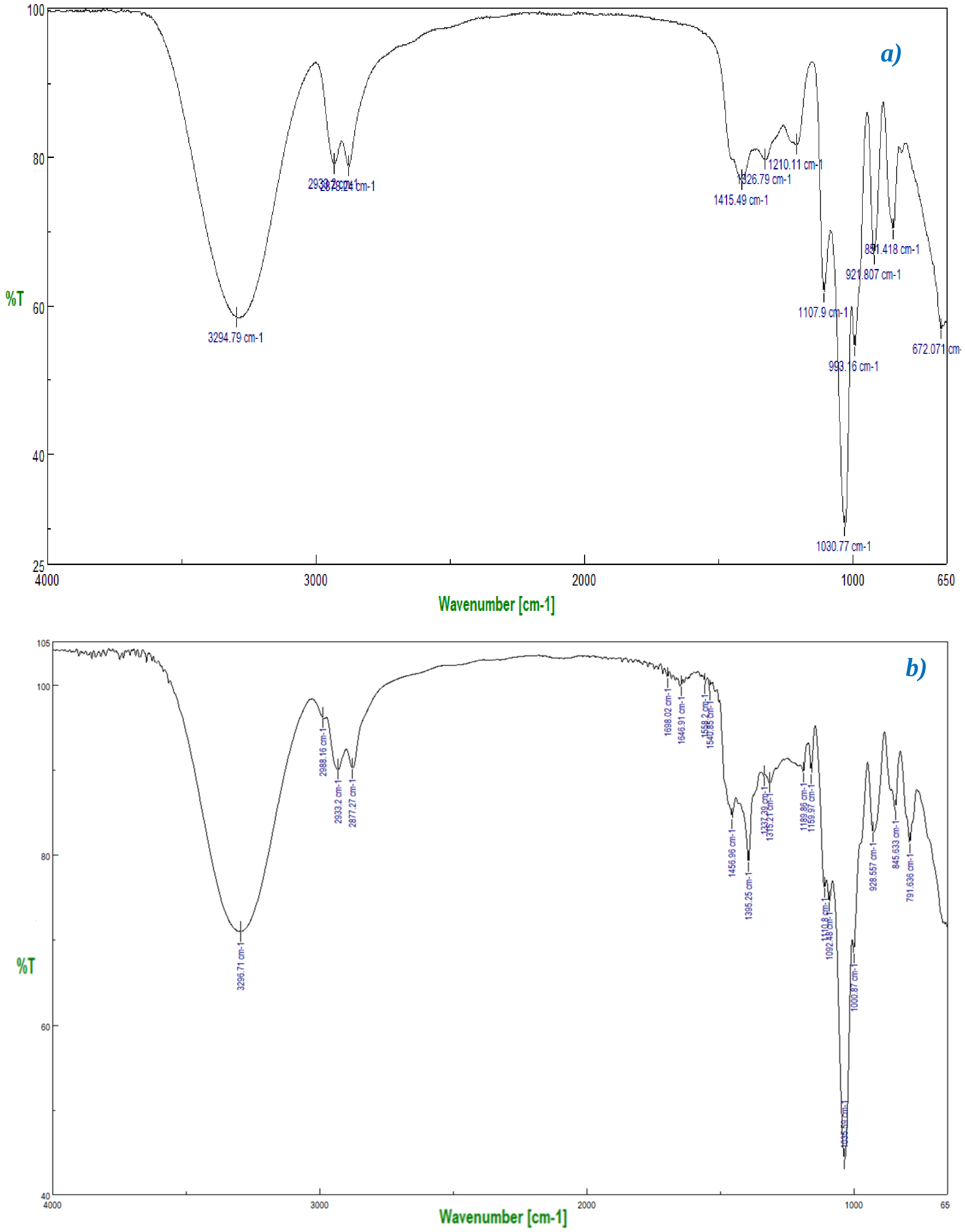

Figure S5: a) FT-IR spectra of glycerol. **b)** FT-IR spectra of [DPTAC][GLY].

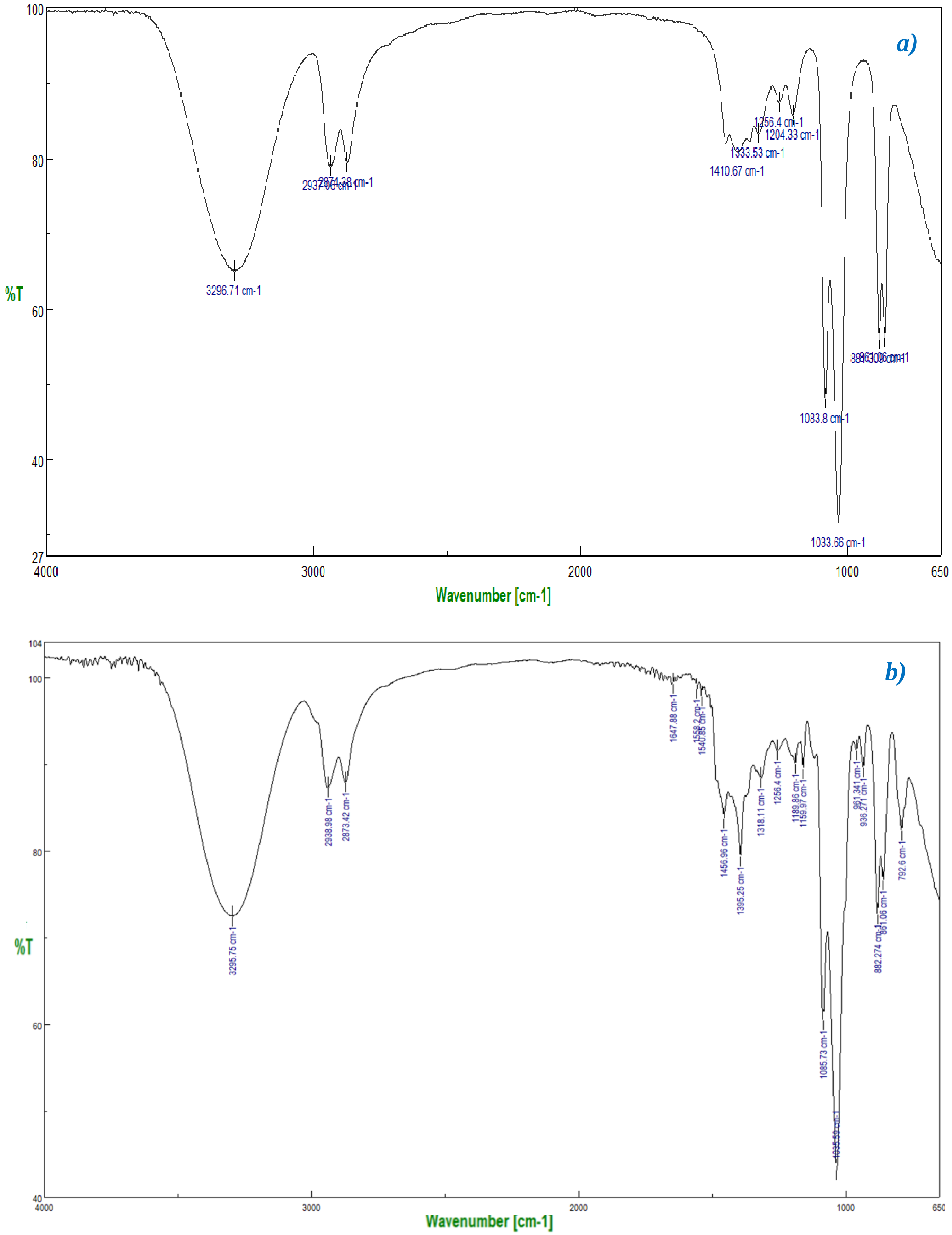

Figure S6: a) FT-IR spectra of ethylene glycol. **b)** FT-IR spectra of [DPTAC][EG]

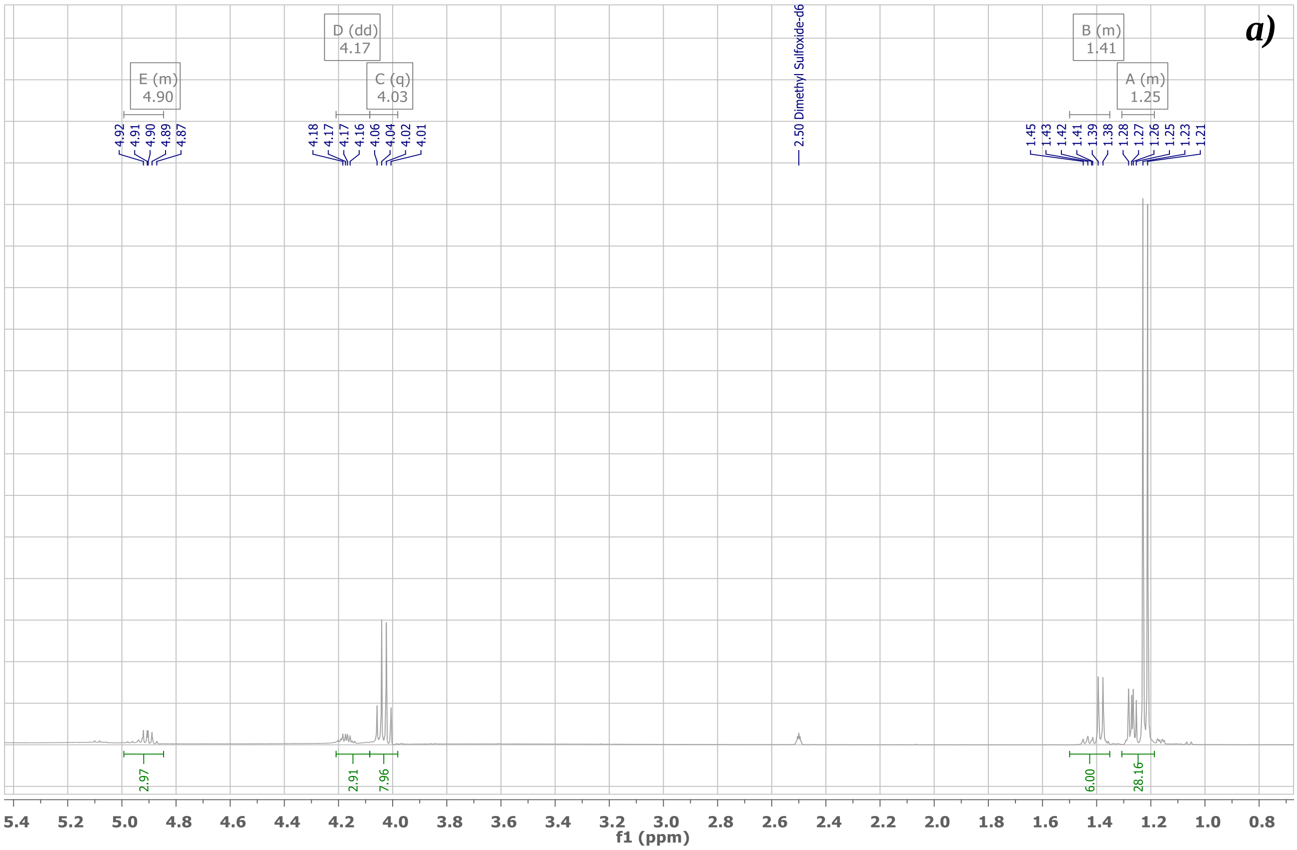

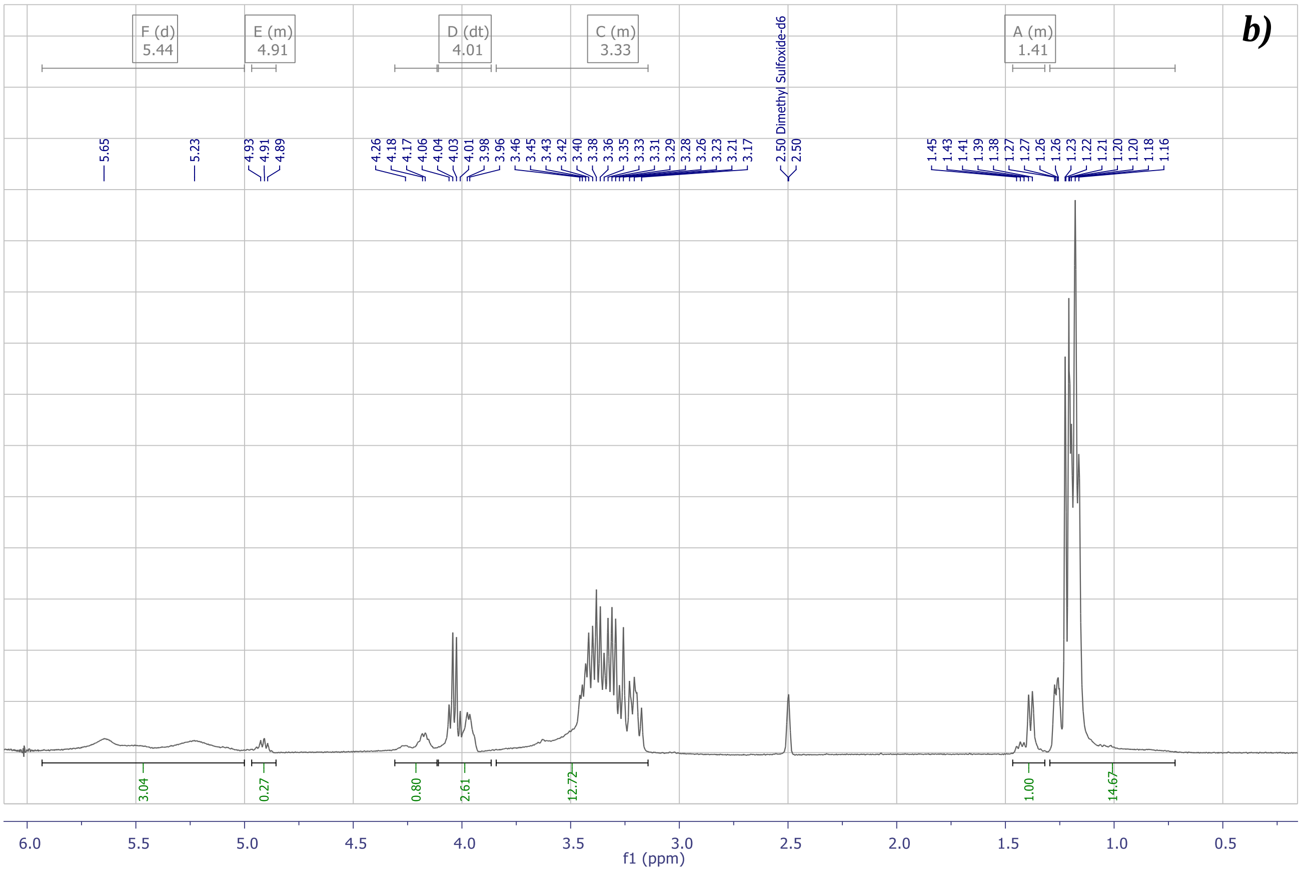

Figure S7: a) ^{1}H NMR (Dmso d_6, 400 MHz) spectra of lactic acid. **b)** ^{1}H NMR (Dmso d_6, 400 MHz) spectra of [DPTAC][LA].

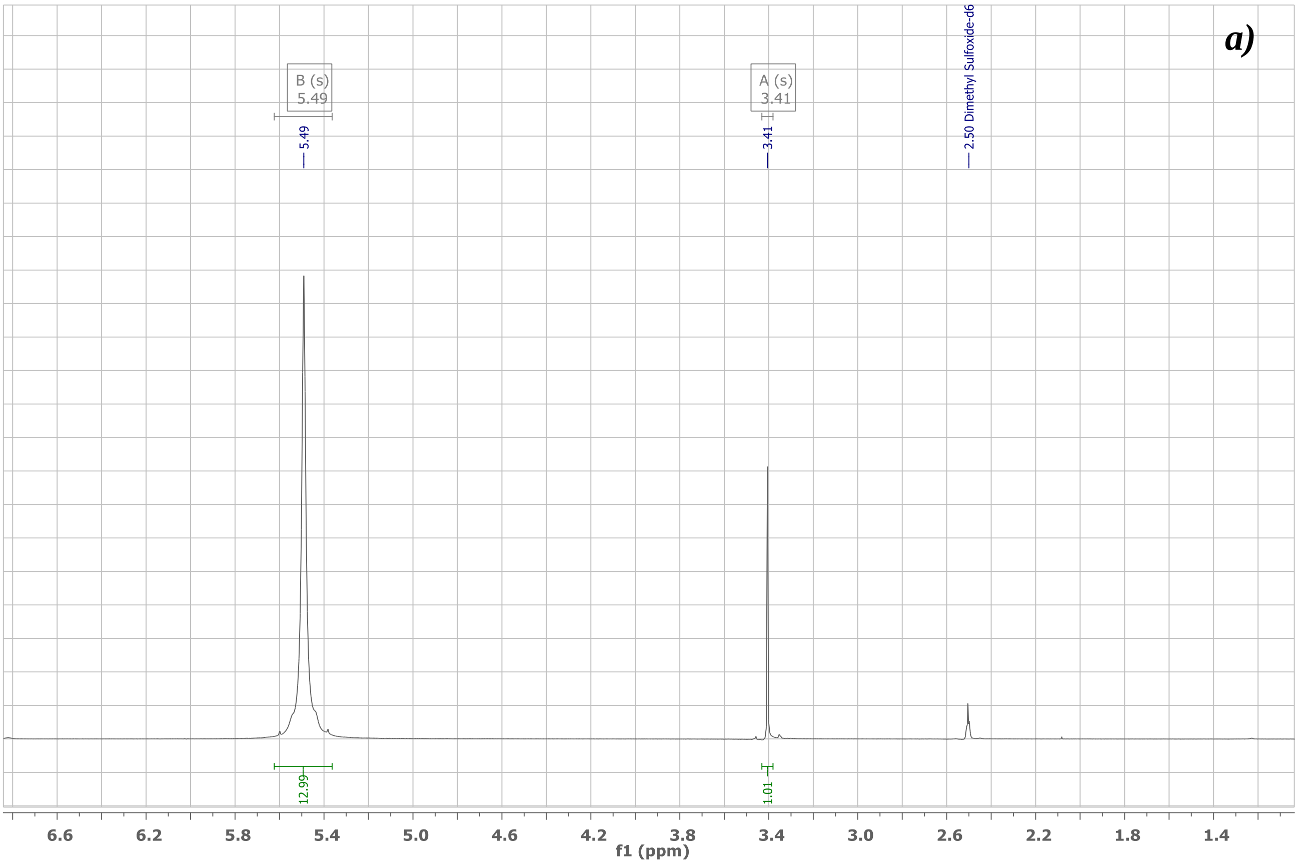

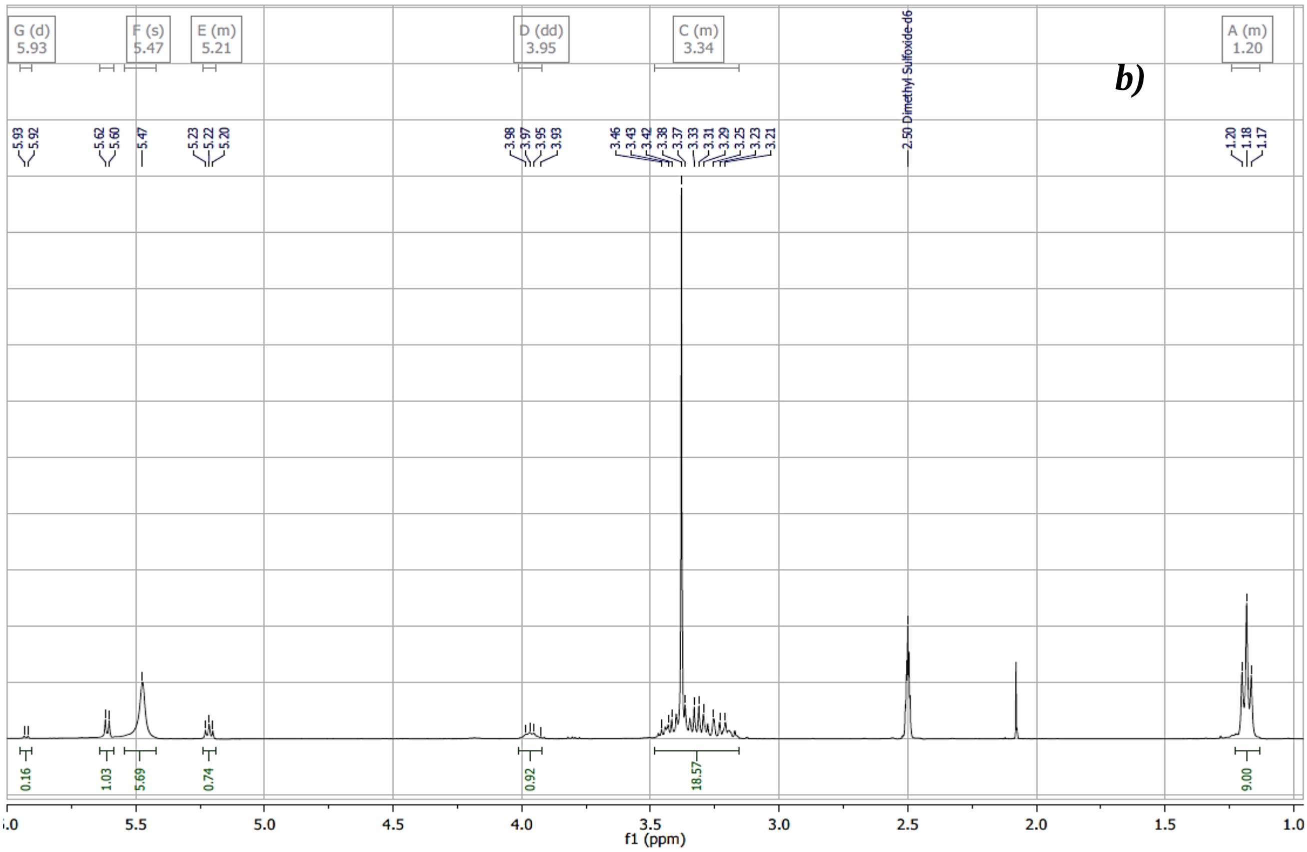

Figure S8: a) ^{1}H NMR (Dmso d_6, 400 MHz) spectra of urea. **b)** ^{1}H NMR (Dmso d_6, 400 MHz) spectra of [DPTAC][UREA].

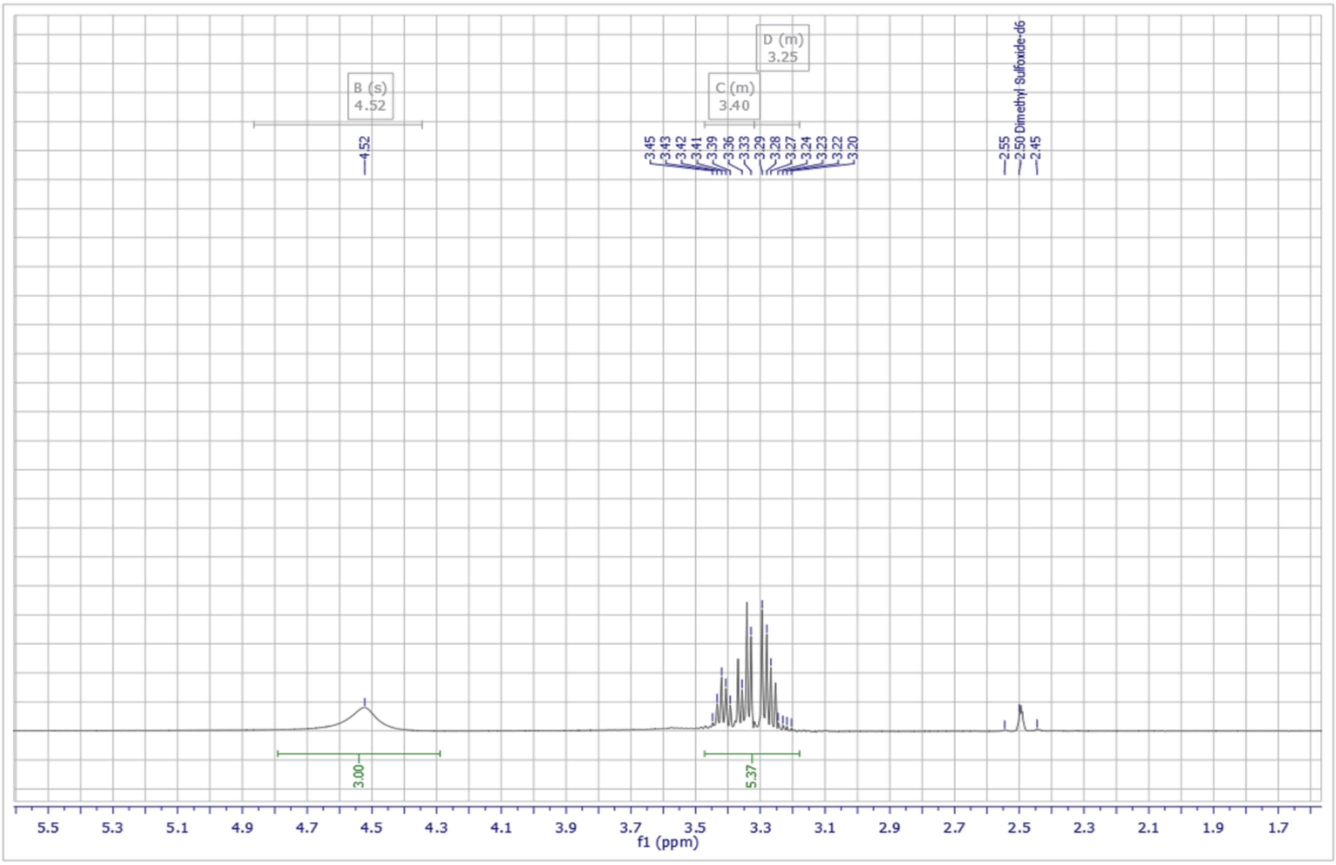

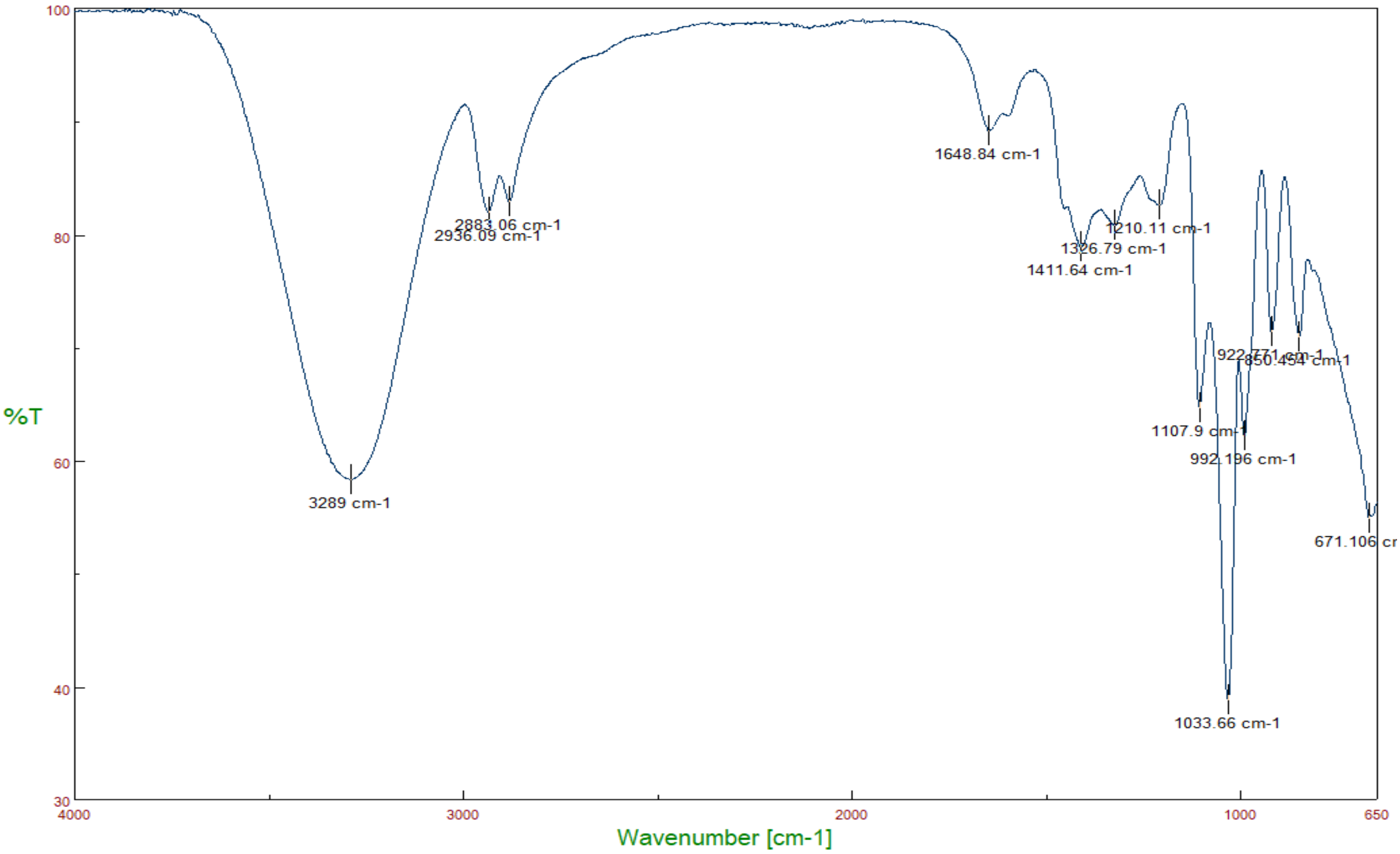

Figure S9: ^{1}H NMR (Dmso d_6, 400 MHz) spectra of glycerol obtained from fat and FT-IR spectra of glycerol obtained from fat.

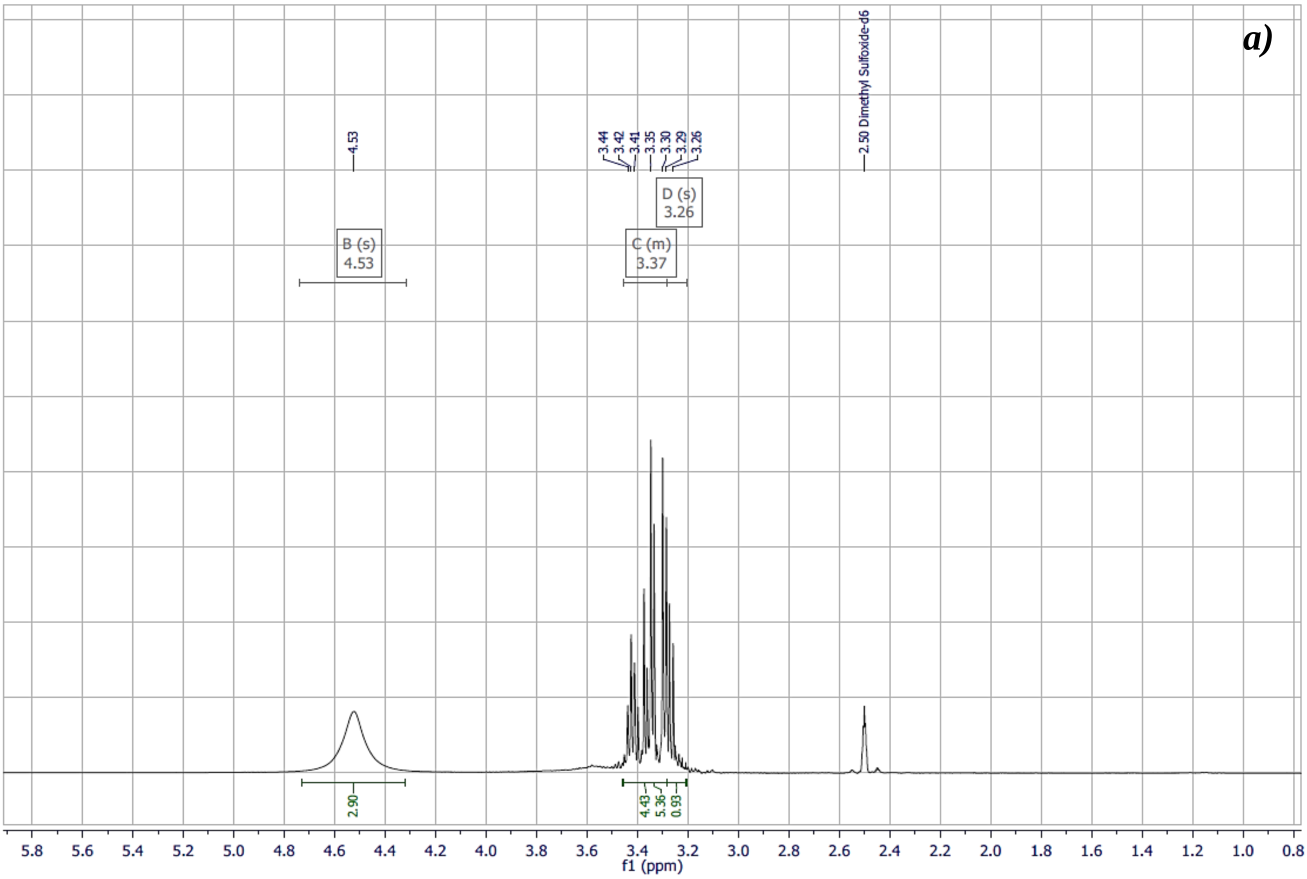

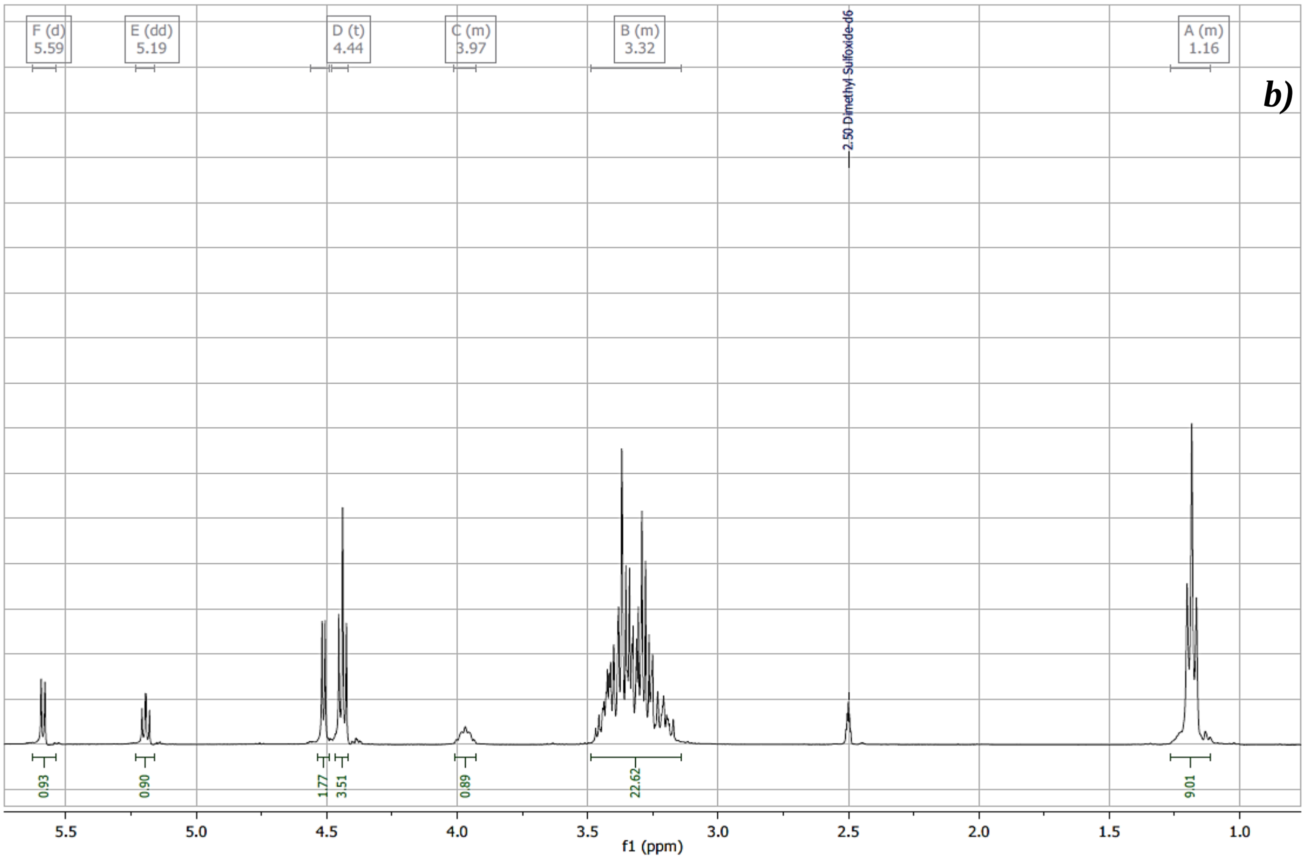

Figure S10: a) ^{1}H NMR (Dmso d_6, 400 MHz) spectra of commercial glycerol. **b)** ^{1}H NMR (Dmso d_6, 400 MHz) spectra of [DPTAC][GLY].

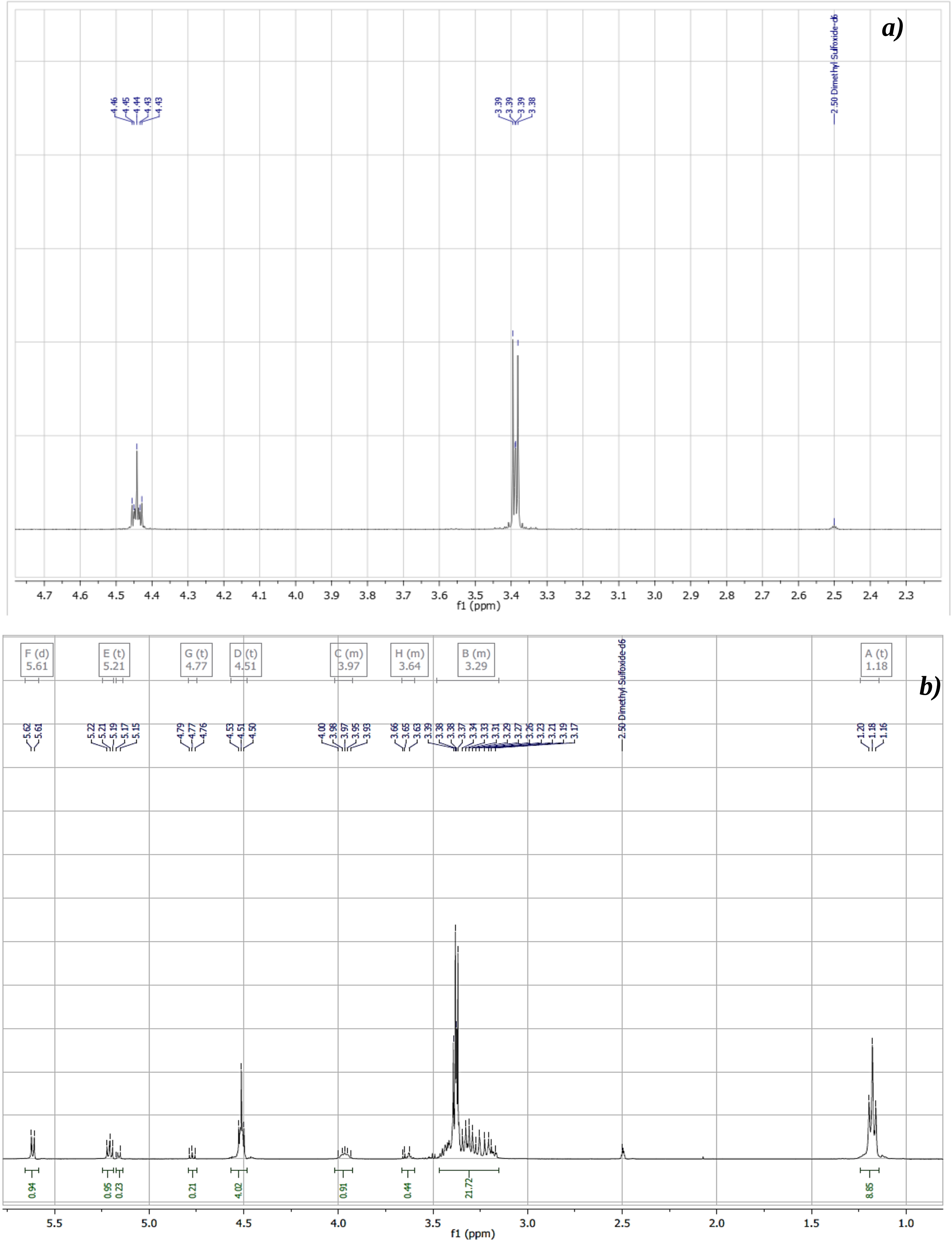

Figure S11: a) ^{1}H NMR (Dmso d_6, 400 MHz) spectra of ethylene glycol. **b)** ^{1}H NMR (Dmso d_6, 400 MHz) spectra of [DPTAC][EG].

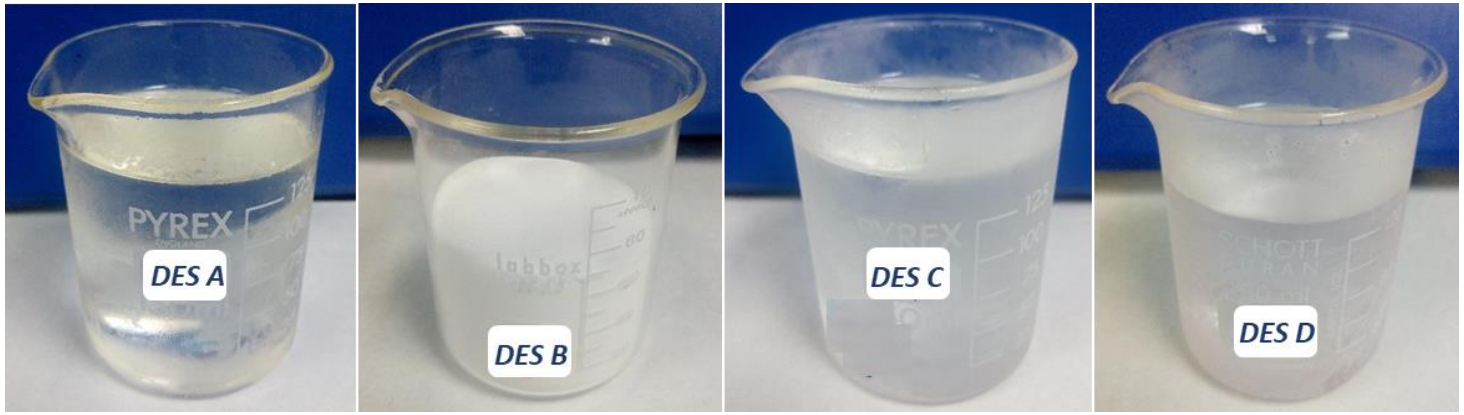

[DPTAC][LA] **[DPTAC]Urea]** **[DPTAC][Gly]** **[DPTAC][Eg]**

Figure S12: DESs obtained between different hydrogen bond donors with the HBA [1].

Table S1: Peak assignments of the ^{1}H-NMR spectrum of 2 before and after the formation of each of the eutectic mixtures.

Before eutectic mixture			After eutectic mixture											
**2**			**[DPTAC][LA]**			**[DPTAC][Urea]**			**[DPTAC][Gly]**			**[DPTAC][Eg]**		
**δ (ppm)**	**shape**	**assignments**	**δ (ppm)**	**shape**	**assignments**	**δ (ppm)**	**shape**	**assignments**	**δ (ppm)**	**shape**	**assignments**	**δ (ppm)**	**shape**	**assignments**
1.18	t	9H	1.26	m	12H	1.20	t	9H	1.16	t	9H	1.18	T	9H
3.03	q	1H	3.34	m	10H	3.34,	m	18H	3.32	m	22H	3.29	m	22H
3.25	dd	1H	4.02	m	2H	3.95	dd	1H	3.97	m	1H	3.64	m	1H
3.39	m	3H	4.18	m	1H	5.21	t	1H	4.44	t	3H	3.97	m	1H
3.48	dd	3H	4.91	m	1H	5.51	bs	4H	4.51	d	1H	4.51	t	4H
3.56	m	1H										4.77	t	1H
3.63	q	1H												
3.96	m	1H												
5.28	t	OH	5.23	bs	OH	5.61	d	OH	5.19	dd	OH	5.21	t	OH
5.74	d	OH	5.65	bs	OH	5.93	d	OH	5.59	d	OH	5.61	d	OH

Table S2: Band assignments of the FT-IR of 2 before and after the formation of each of the eutectic mixtures.

Before eutectic mixture		After eutectic mixture							
**2**		**[DPTAC][LA]**		**[DPTAC][Urea]**		**[DPTAC][Gly]**		**[DPTAC][Eg]**	
**cm^{-1}**	**group**	**cm^{-1}**	**group**	**cm^{-1}**	**group**	**cm^{-1}**	**group**	**cm^{-1}**	**group**
3284.18	(OH)	3331.18	(OH)	3426.89	(N-H)	3296.71	(OH)	3295.75	(OH)
3216.68	(OH)	2986.23	(CH_3)	3326.61	(N-H)	2933.20	(CH_2)	2938.98	(CH)
2986.23	(C-H alkyl)	1456.96	(CH_3)	3255.25	$(N-H_2)$	2877.21	(N^+-CH)	2873.42	(CH)
2924.52	(C-H alkyl)	1372.10	(CH_3)	1154.19	$(N-H_2)$	1456.96	(CH_2)	1456.96	(CH_2)
2885.92	(N^+CH)	1729.83	(C=O)	1672.95	(C=O)	1395.25	$(N-CH_3)$	1395.25	$(N-CH_3)$
2817.49	(N^+CH)	1203.36	(C-O)	1457.92	(C-N)	1337.39	(CH_2)	1256.40	(CH_2)
1489.74	$((CH_2)^3-N^+)$	1124.30	(C-O)	1089.58	(C-N)	1159.97	(C-N)	1159.97	(C-N)
1398.14	(CH_2-N)	1042.34	(C-O)	1001.84	(C-N)	1092.48	(C-N)	1085.73	(-C-O)
1373.07	(C-N)			786.81	(H_2N-CO)	1110.80	(C-O)	1035.59	(O-C-C-O)
1292.07	(C-N)					1035.59	(C-O)	882.27	(CH_2)
1163.83	(C-N)					1000.87	$((CH_2)_3-N^+)$	861.06	(C-C)
1087.66	(C-N)					928.55	(C-OH)		
1150.83	(C-O-C)					845.63	$(-O-C_2H_4)$		
1040.00	(CO)								
1002.8	$((CH_2)_3-N^+)$								
961.34	(C-C)								
937.23	(C-C)								
847.56	(CH)								
793.56	$(\gamma\ CH_2)$								
703.89	(CH_2)								

Table S3: Total content of lignocellulosic material in dry samples.

**Sample**	**% hemicellulose**	**% cellulose**	**% lignin**
Apricot	27	37	14
Plum	10	41	26
Peach	10	40	18
Nectarine	10	41	16
Flat Peach	11	36	28
Olive Pomace	3,4	16	37

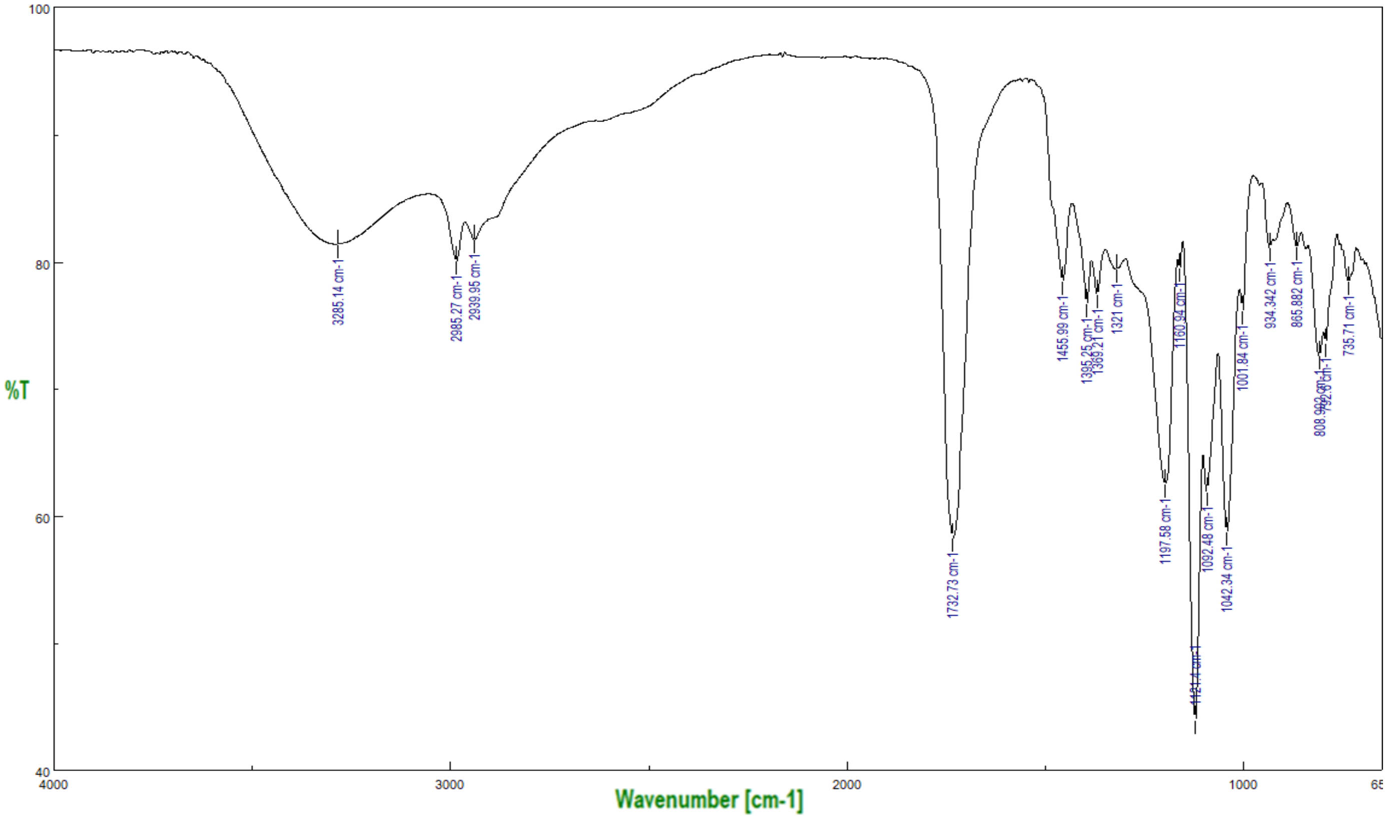

Figure S13: FT-IR spectra of “filtrate 2” fraction from olive pomace using [DPTAC][LA].

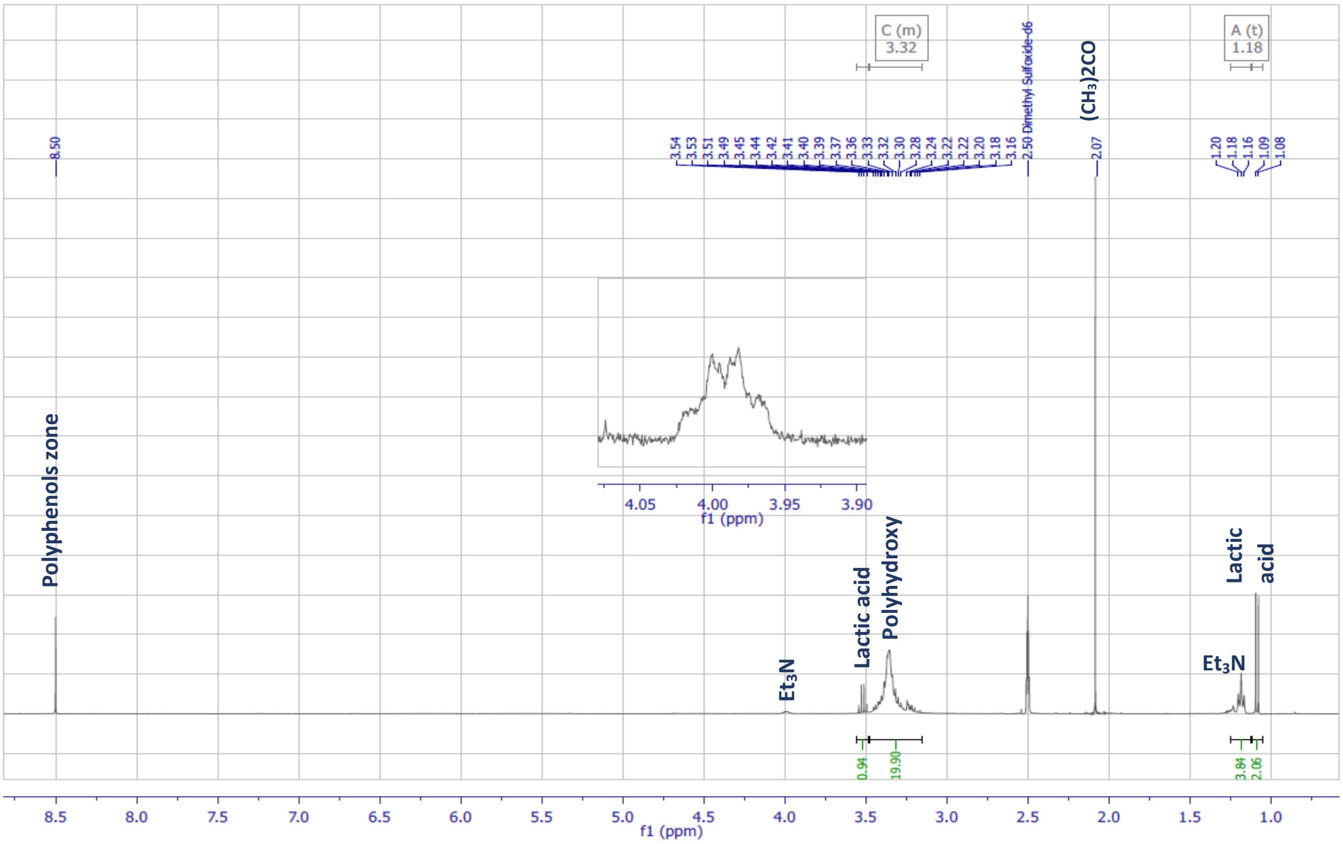

Figure S14: ^{1}H NMR (Dmso d_6, 400 MHz) spectra of “filtrate 3” fraction from olive pomace using [DPTAC][LA].

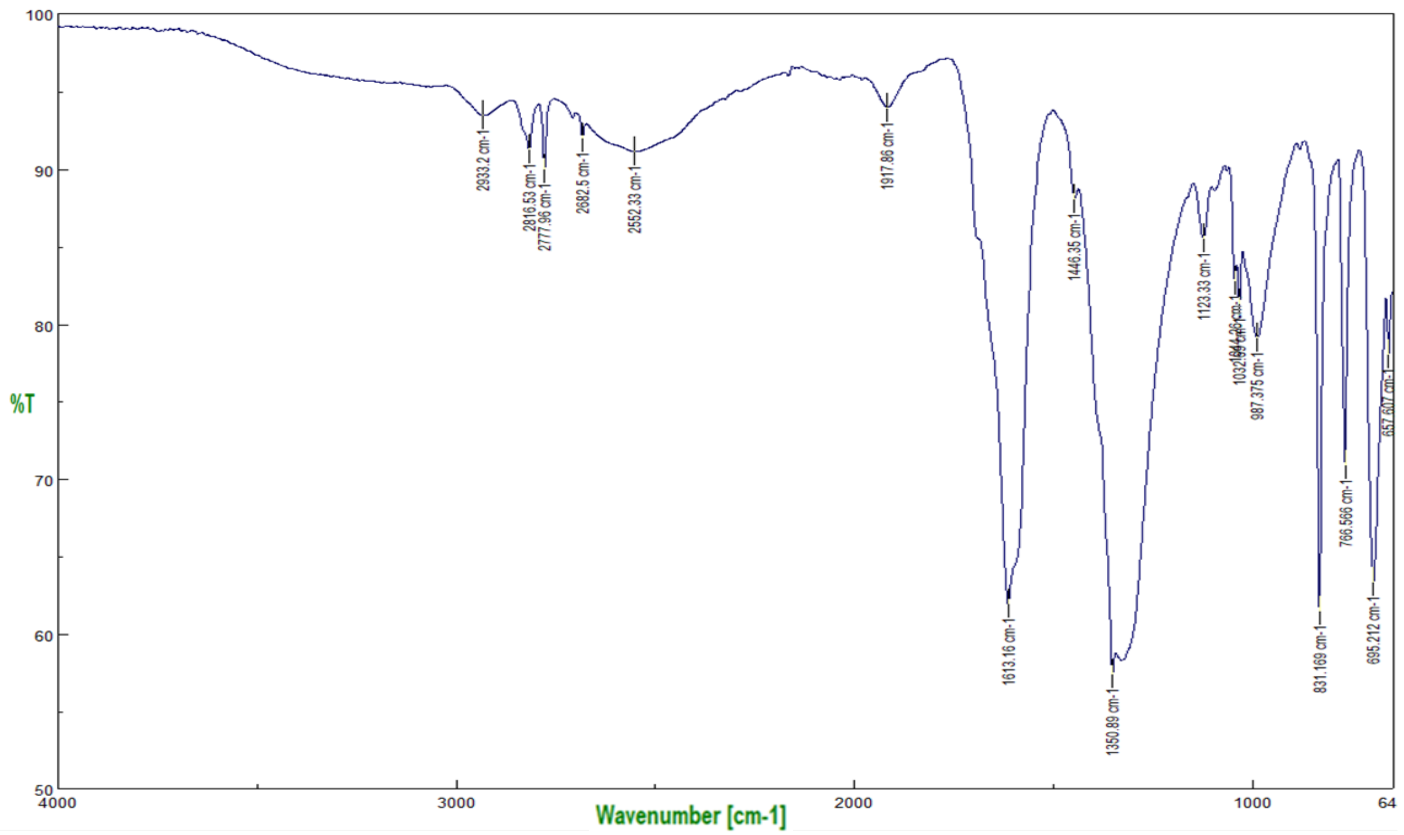

Figure S15: FT-IR spectra of “filtrate 3” fraction from olive pomace using [DPTAC][LA].

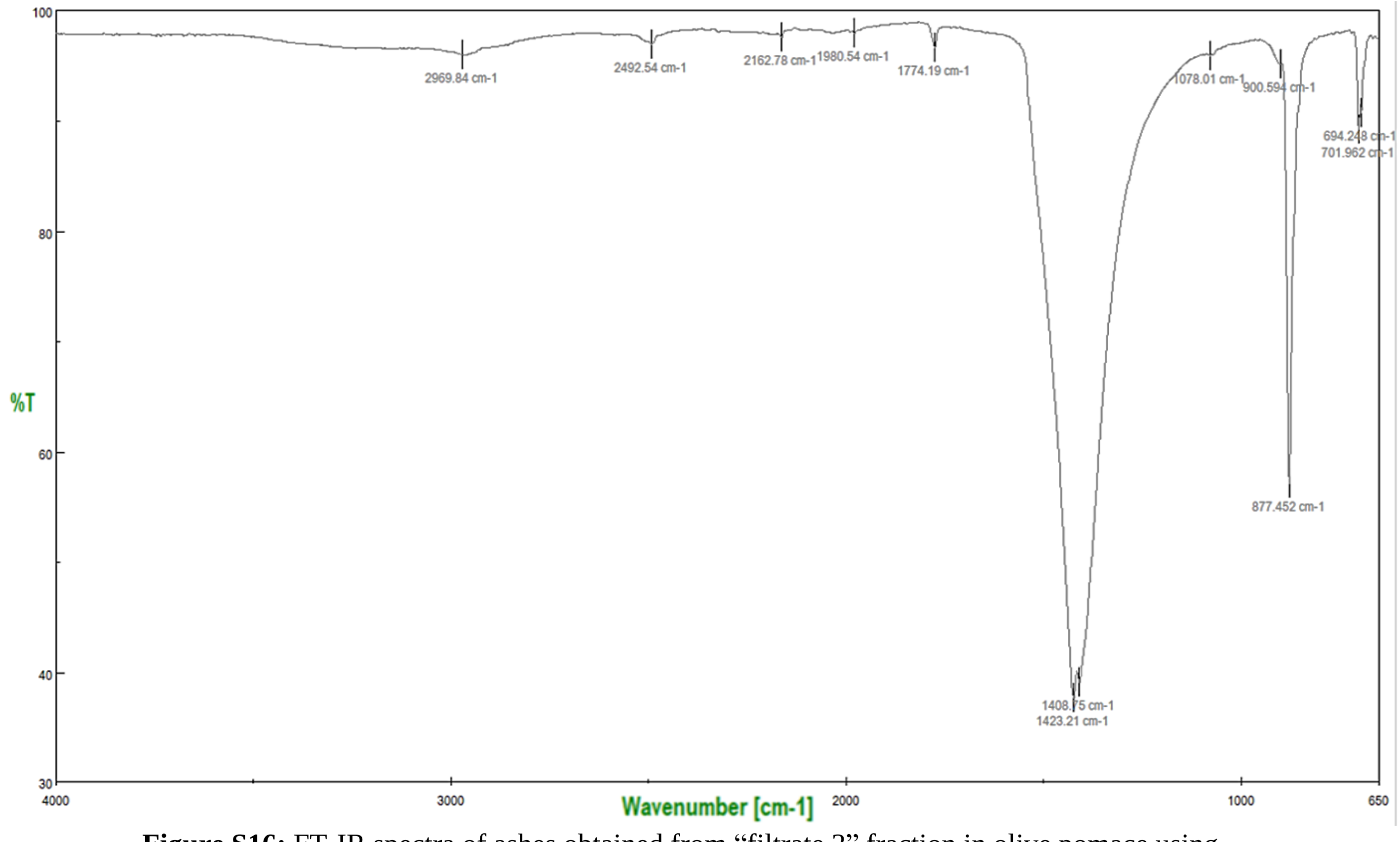

Figure S16: FT-IR spectra of ashes obtained from “filtrate 3” fraction in olive pomace using [DPTAC][LA].

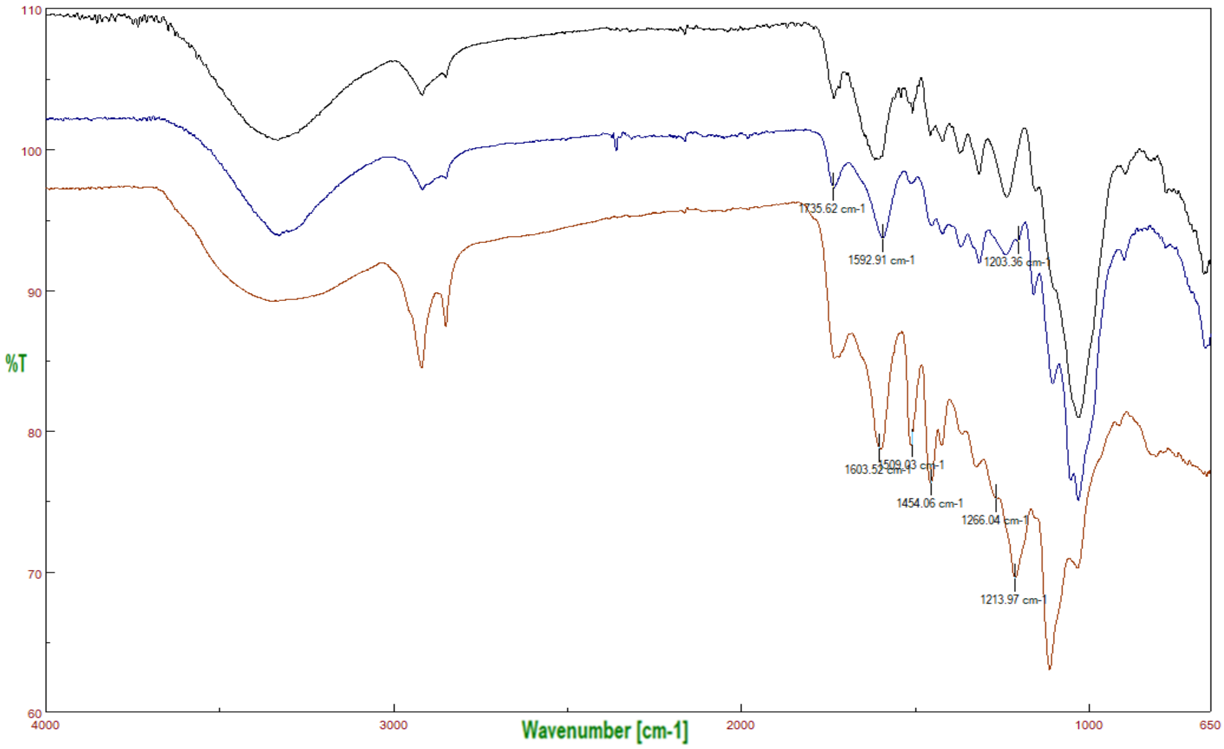

Figure S17: FT-IR spectra of pruning waste of apricot branches tree before treatment (black line), holocellulose-rich fraction (blue line) and extracted lignin (red line) using [DPTAC][LA].

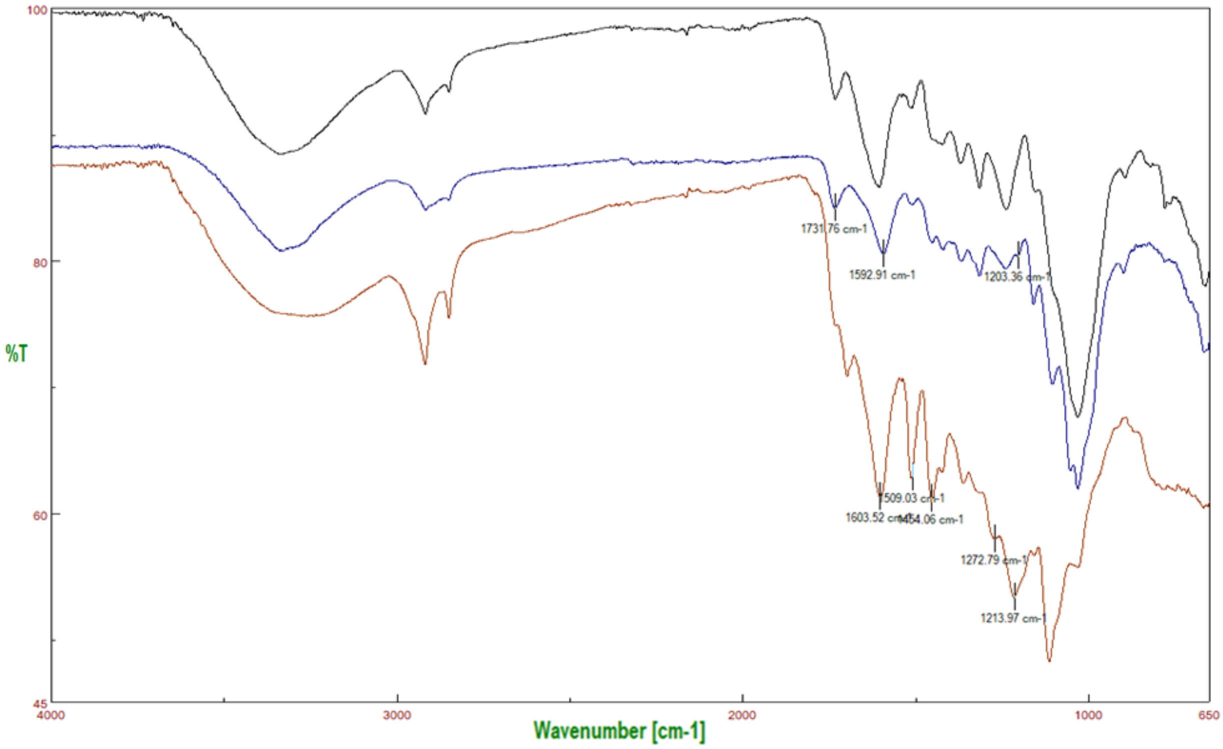

Figure S18: FT-IR spectra of pruning waste of plum branches tree before treatment (black line), holocellulose-rich fraction (blue line) and extracted lignin (red line) using [DPTAC][LA].

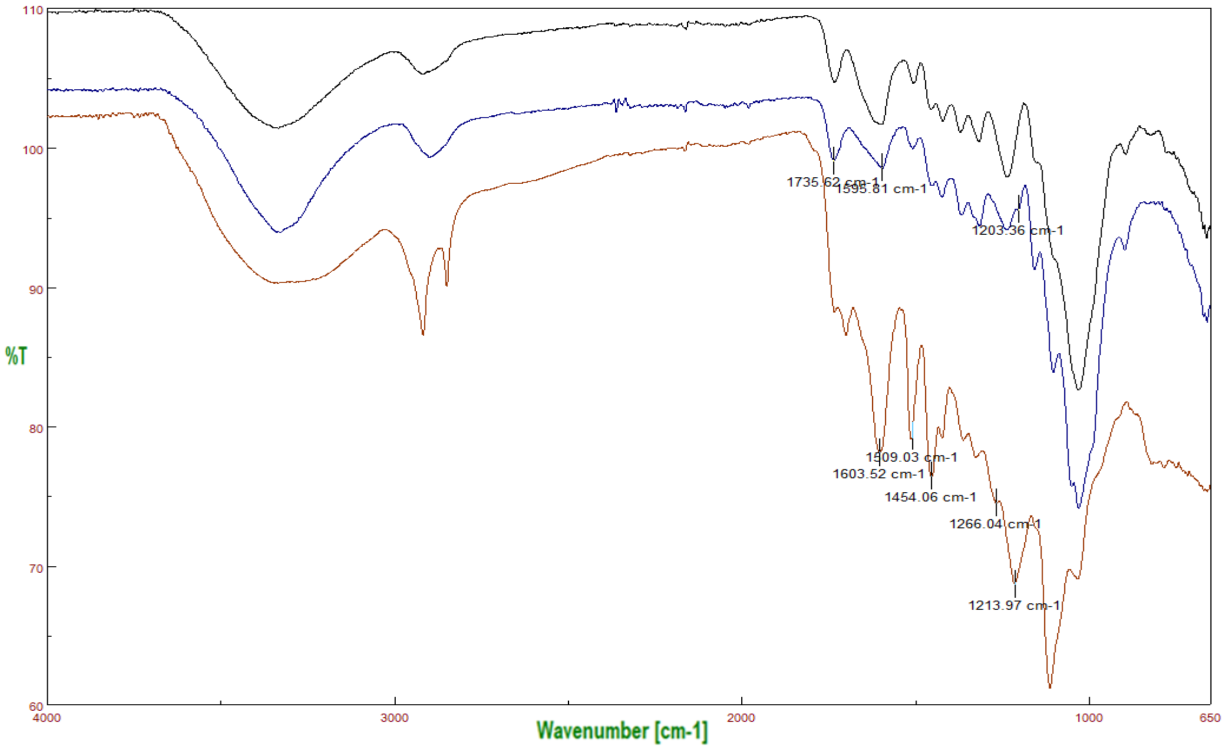

Figure S19: FT-IR spectra of pruning waste of peach branches tree before treatment (black line), holocellulose-rich fraction (blue line) and extracted lignin (red line) using [DPTAC][LA].

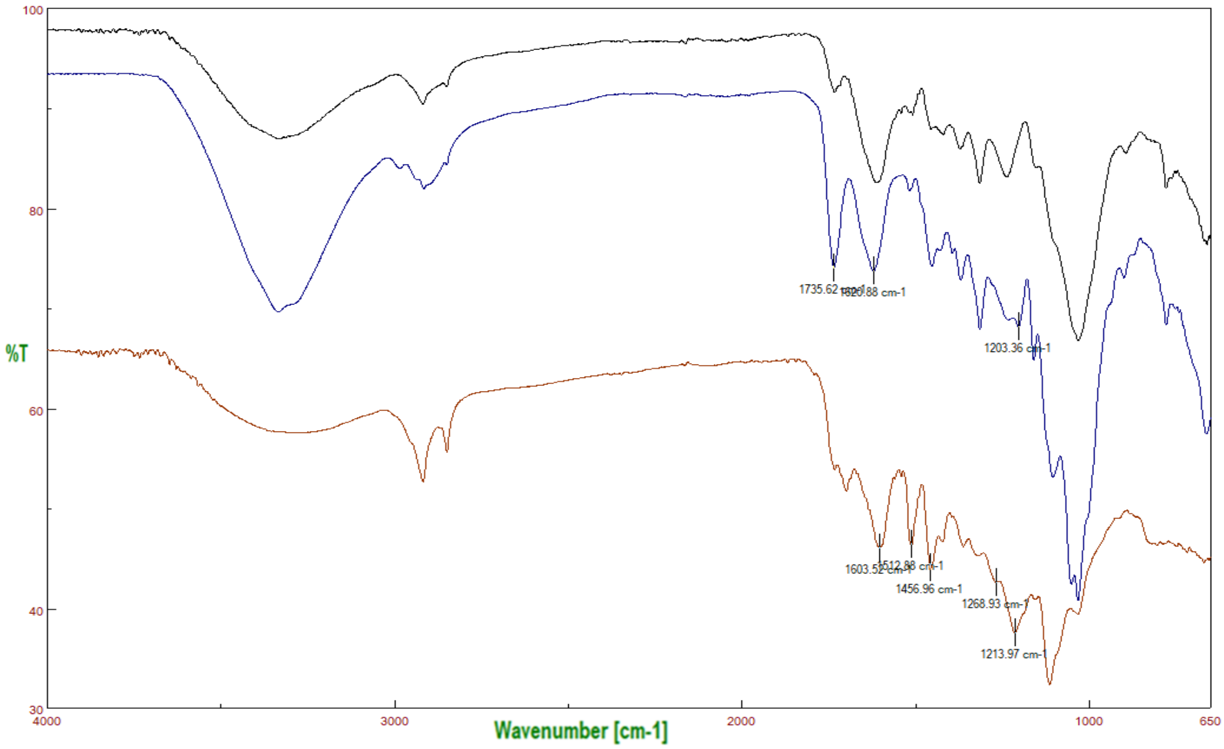

Figure S20: FT-IR spectra of pruning waste of nectarine branches tree before treatment (black line), holocellulose-rich fraction (blue line) and extracted lignin (red line) using [DPTAC][LA].

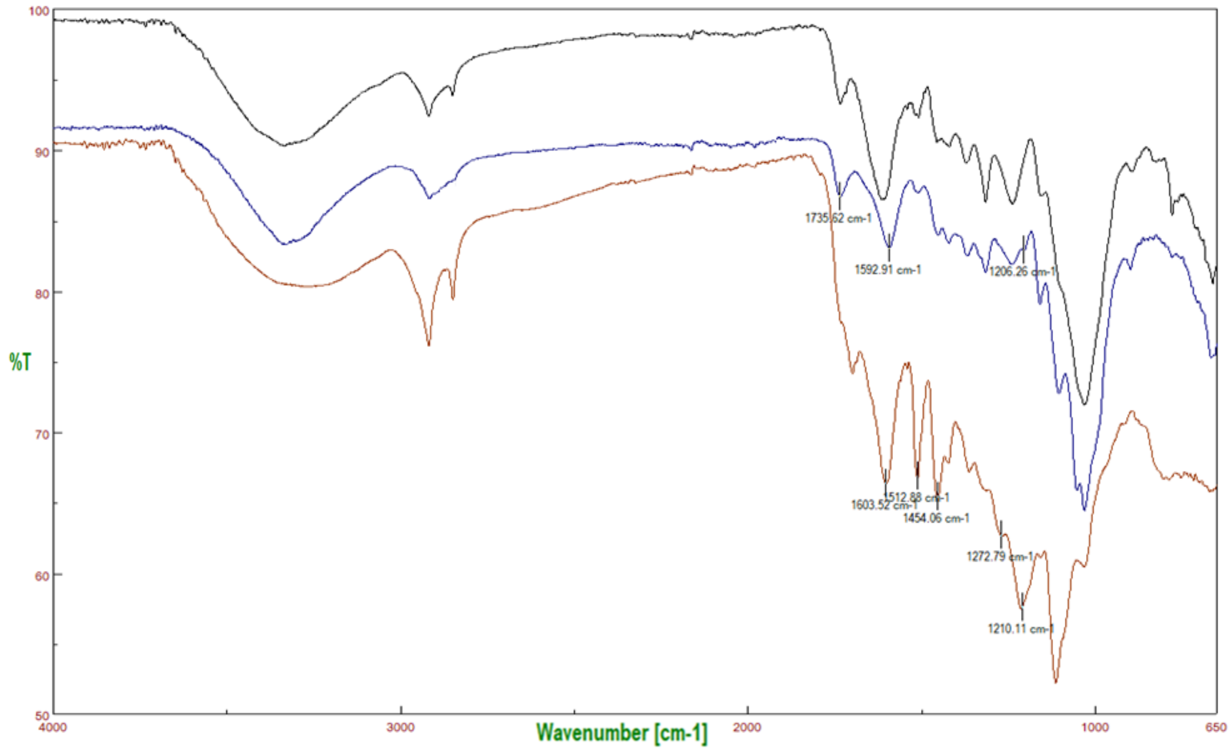

Figure S21: FT-IR spectra of pruning waste of flat peach branches tree before treatment (black line), holocellulose-rich fraction (blue line) and extracted lignin (red line) using [DPTAC][LA].

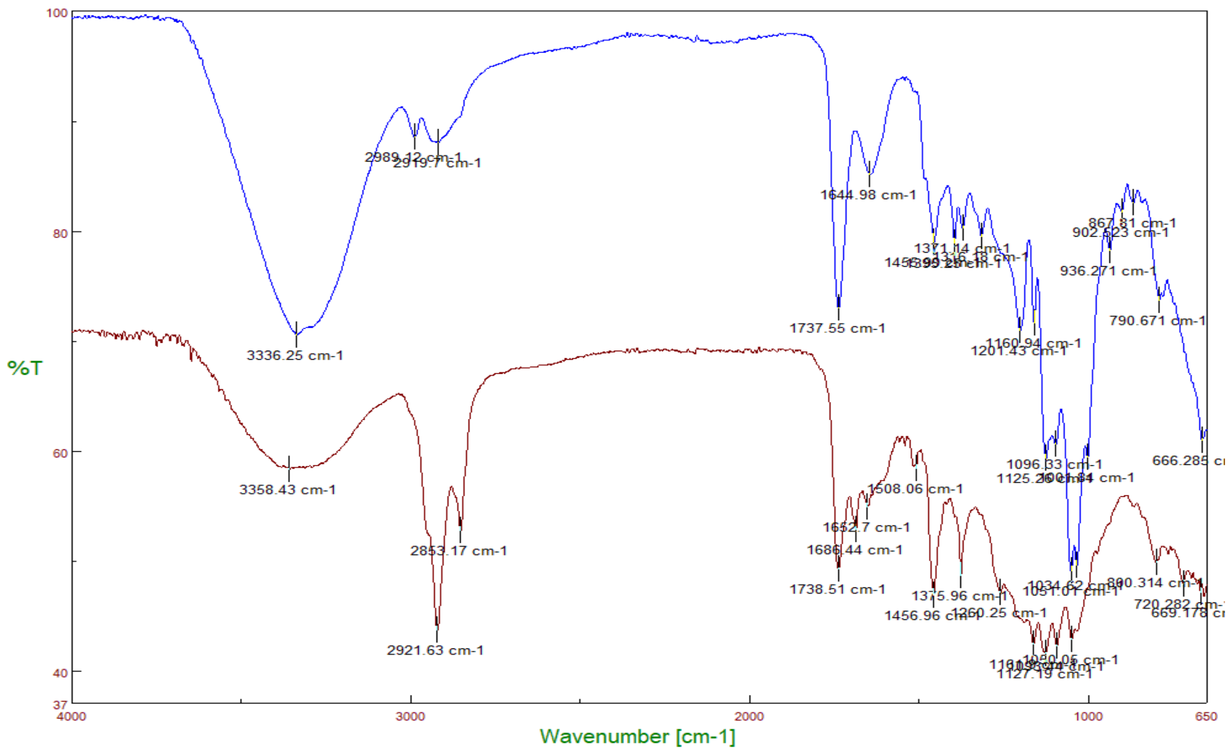

Figure S22: FT-IR spectra of holocellulose-rich fraction (blue line) and lignin (red line) obtained from olive pomace using [DPTAC][LA] extracted at 150°C.

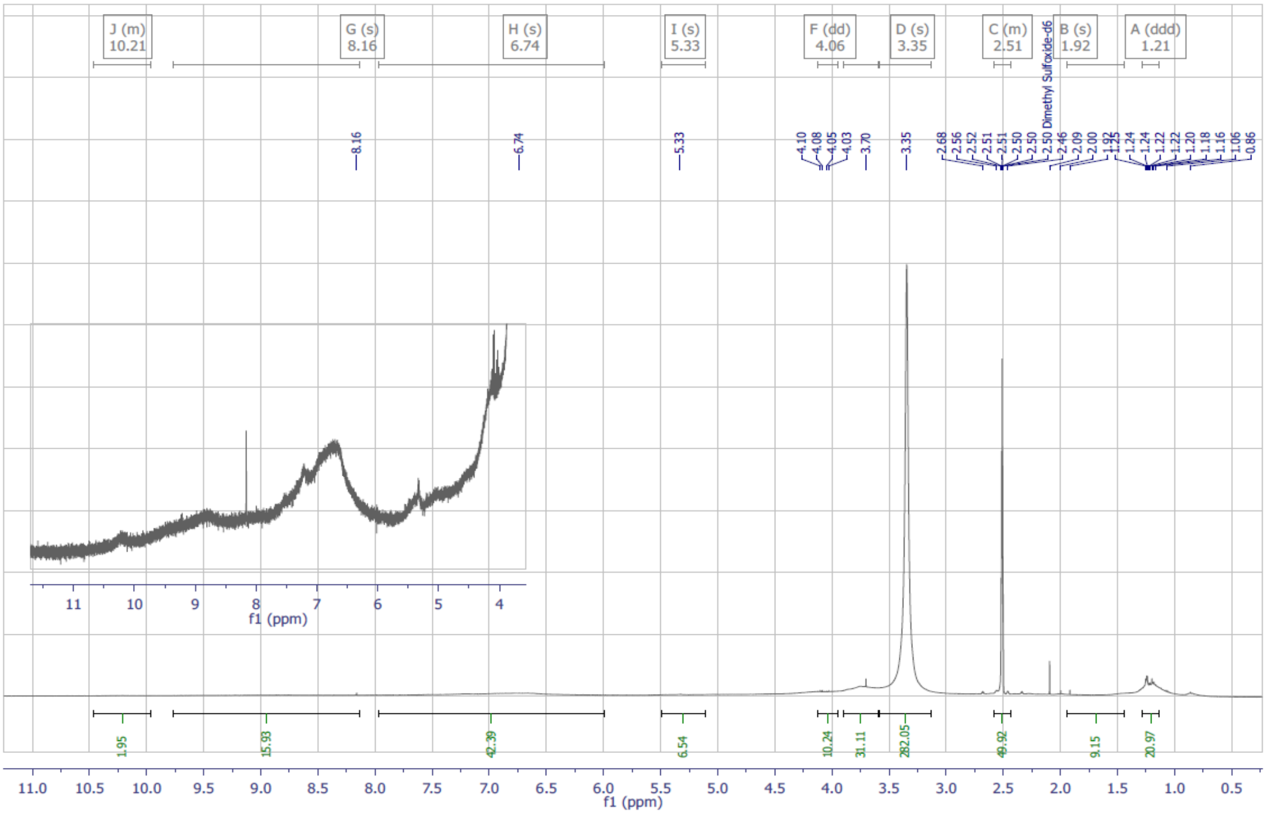

Figure S23. ^{1}H NMR (Dmso d_6, 400 MHz) spectra of lignin fraction obtained from olive pomace using [DPTAC][LA].

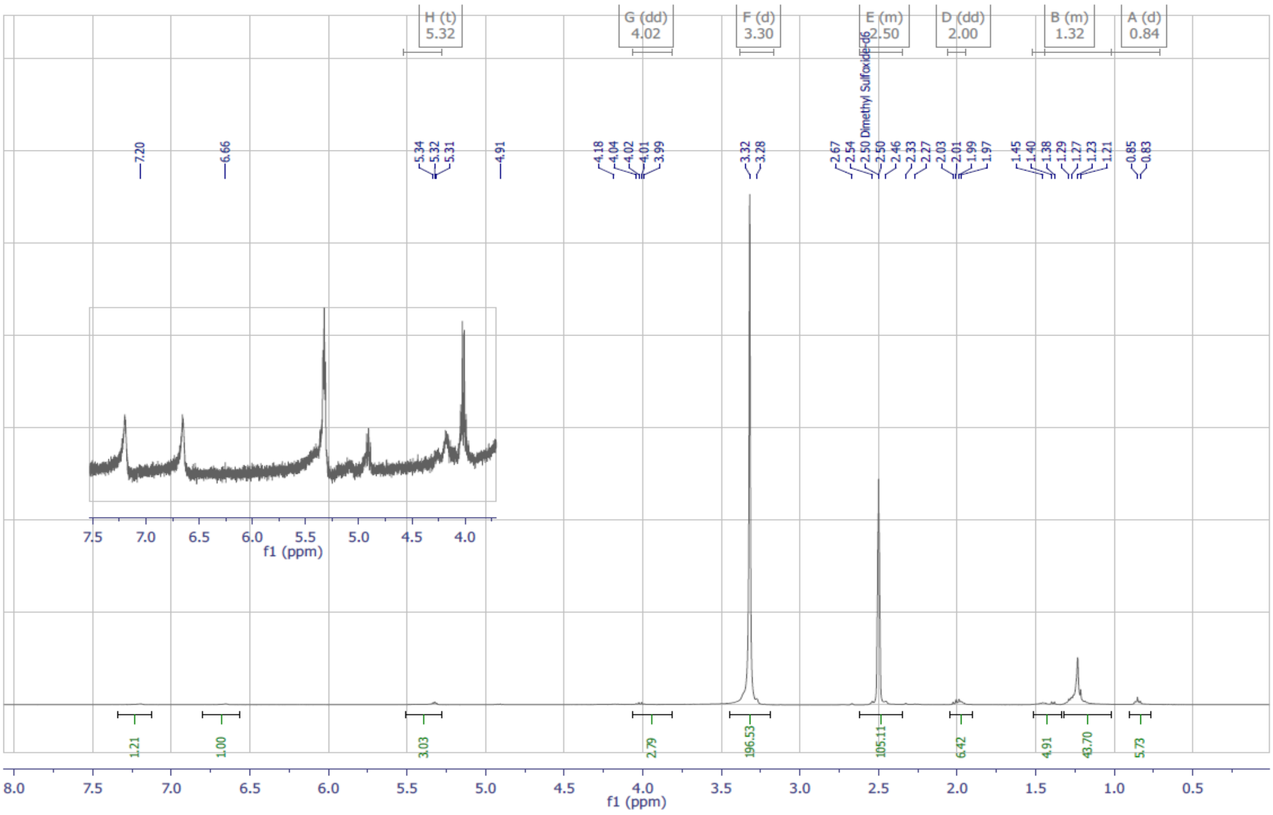

Figure S24: ^{1}H NMR (Dmso d_6, 400 MHz) spectra of lignin fraction obtained from olive pomace using [DPTAC][UREA].

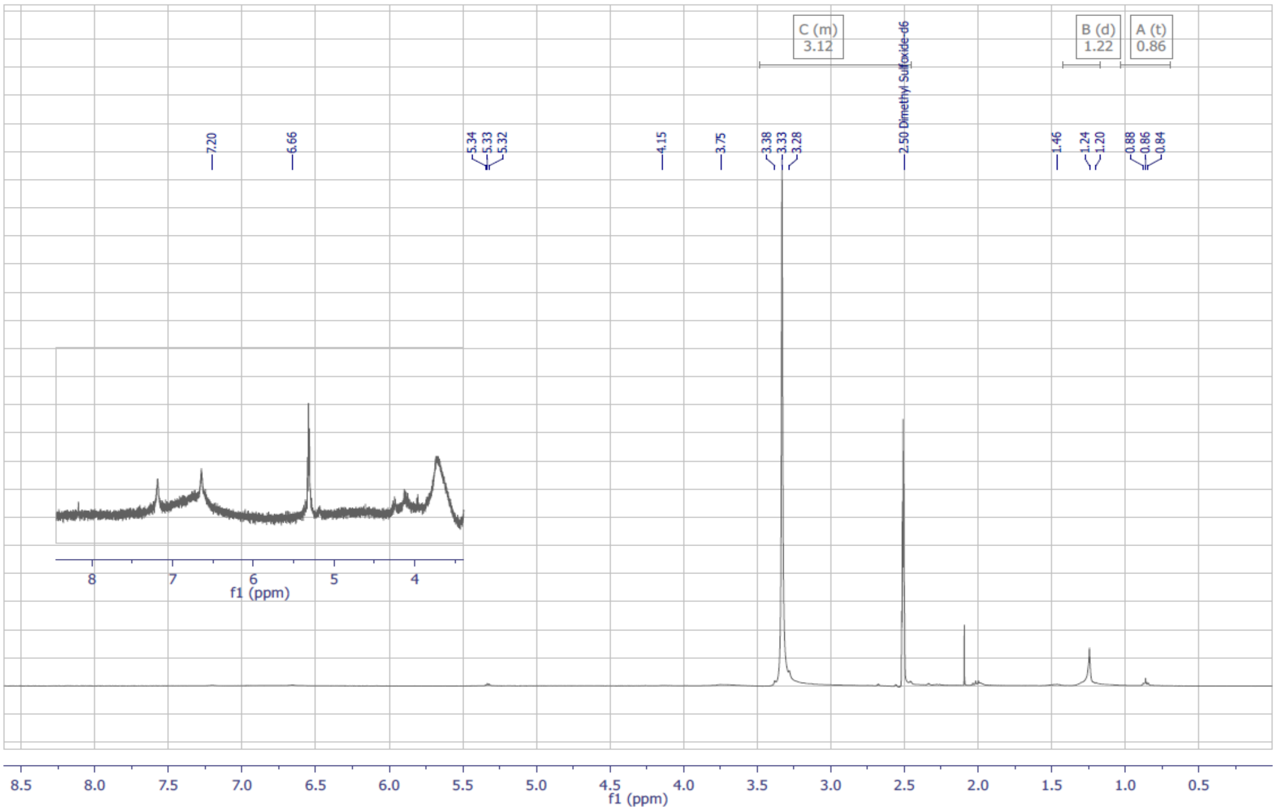

Figure S25: ^{1}H NMR (Dmso d_6, 400 MHz) spectra of lignin fraction obtained from olive pomace using [DPTAC][GLY].

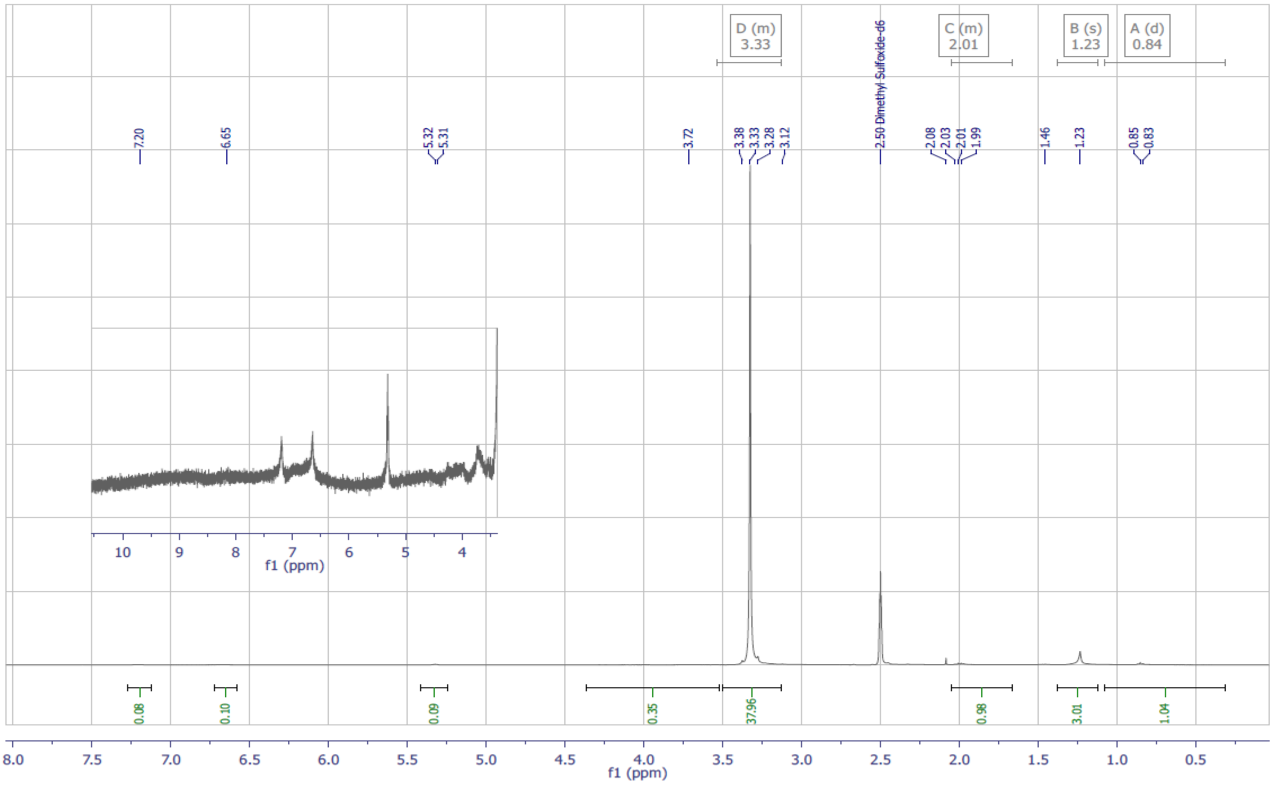

Figure S26: ^{1}H NMR (Dmso d_6, 400 MHz) spectra of lignin fraction obtained from olive pomace using [DPTAC][EG].

Table S4: Molecular weight (Mw) and polydispersity index (PDI) of lignin determined by GPC (Gel permeation chromatography) in hardwood samples

Sample	DES/IL	Mw	PDI
Apricot	[DPTAC][LA].	30986	2,4
Plum	[DPTAC][LA].	45192	2,5
Peach	[DPTAC][LA].	57771	2,6
Nectarine	[DPTAC][LA].	39955	2,3
Flat Peach	[DPTAC][LA].	41979	2,3
Olive Pomace	[DPTAC][LA].	20486	2,4
Olive Pomace	IL	113677	6,3
